# Talaroterpenoids A–F: Six New *Seco*-Terpenoids from the Marine-Derived Fungus *Talaromyces aurantiacus*

**DOI:** 10.3390/md22100475

**Published:** 2024-10-18

**Authors:** Zi-Hong Peng, Hui Jia, Yan-Liang Luo, Li-Jun Zhang, Jia-Tong Zhou, Yuan-Han Xie, Li-Jun Wang, Jiang-Ke Qin, Jun Li, Guo-Hai Zhang, Rui-Yun Yang, Wei-Feng Xu

**Affiliations:** 1 State Key Laboratory for Chemistry and Molecular Engineering of Medicinal Resources, Collaborative Innovation Center for Guangxi Ethnic Medicine, School of Chemistry and Pharmaceutical Sciences, Guangxi Normal University, Guilin 541004, China; pengzihong000311@163.com (Z.-H.P.); jiahui18878391665@163.com (H.J.); lyl2010927@163.com (Y.-L.L.); ajun840618@mailbox.gxnu.edu.cn (L.-J.Z.); zjt990123@163.com (J.-T.Z.); xieyuanhan0330@163.com (Y.-H.X.); jiangke@sina.com (J.-K.Q.); lijun9593@gxnu.edu.cn (J.L.); 2 School of Design, Guangxi Normal University, Guilin 541004, China; gxsd307@126.com

**Keywords:** *seco*-terpenoids, *Talaromyces aurantiacus*, anti-inflammatory activities, marine-derived fungus

## Abstract

Six new highly oxidized *seco*-terpenoids, including three 3-*nor*-labdane type diterpenes, talaroterpenoids A–C (**1**–**3**), and three meroterpenoids containing an orthoester group, talaroterpenoids D–F (**6**–**8**), together with five known compounds (**4**–**5** and **9**–**11**), were isolated from the marine-derived fungus *Talaromyces aurantiacus*. Their chemical structures were elucidated through 1D, 2D NMR, HRESIMS, *J*-based configuration analysis (JBCA), computational ECD calculations, and single-crystal X-ray diffraction analysis. Compounds **1** and **2** contain an unusual 6,20-*γ*-lactone-bridged scaffold. Compounds **10** and **11** presented inhibitory effects on NO release in lipopolysaccharide (LPS)-induced BV-2 cells with IC_50_ values of 11.47 and 11.32 μM, respectively. Talaroterpenoid C (**3**) showed moderate antifungal activity against *A. alternata* and *P. theae* Steyaert.

## 1. Introduction

Marine-derived fungi are recognized as a significant source of chemically diverse and bioactive organic molecules. The vastness and unique environmental conditions of the ocean have fostered the evolution of these fungi, endowing them with the capacity to produce an array of secondary metabolites. To date, over 35,000 marine natural products have been characterized, and 13 marine-derived drugs have been approved for clinical use [1,2,3,4,5,6]. Among them, the genus *Talaromyces* has shown great potential in agriculture, food, cosmetics, medicine, and environmental protection, emerging as a promising source for bioactive compound discovery [7,8,9]. Secondary metabolites isolated from marine-derived *Talaromyces* species exhibited various bioactivities, including anti-inflammatory [10], antibacterial [11], antitumor [12], and antifouling properties [13].

*Seco*-labdanes represent an intriguing class of diterpenoids distinguished by the oxidative cleavage of a carbon–carbon (C–C) bond during their biosynthesis [14]. This biochemical modification diversifies the rigid labdane skeleton, giving rise to a plethora of structurally diverse compounds with increased chemical and biological complexity [15]. This process is often facilitated by enzymes such as Baeyer–Villiger monooxygenases, leading to the formation of *nor*- and *seco*-terpenoids. To date, over 100 *seco*-labdanes have been isolated, and these compounds can be classified into several subclasses based on the specific C–C bond that undergoes cleavage: 2,3-*seco*-labdanes, 3,4-*seco*-labdanes, 6,7-*seco*-labdanes, 7,8-*seco*-labdanes, 8,9-*seco*-labdanes, and 9,10-*seco*-labdanes [16]. Among these subclasses, 2,3-*seco*-labdanes feature a cleavage between the C-2 and C-3 positions of the labdane skeleton [17].

**Figure 1:**
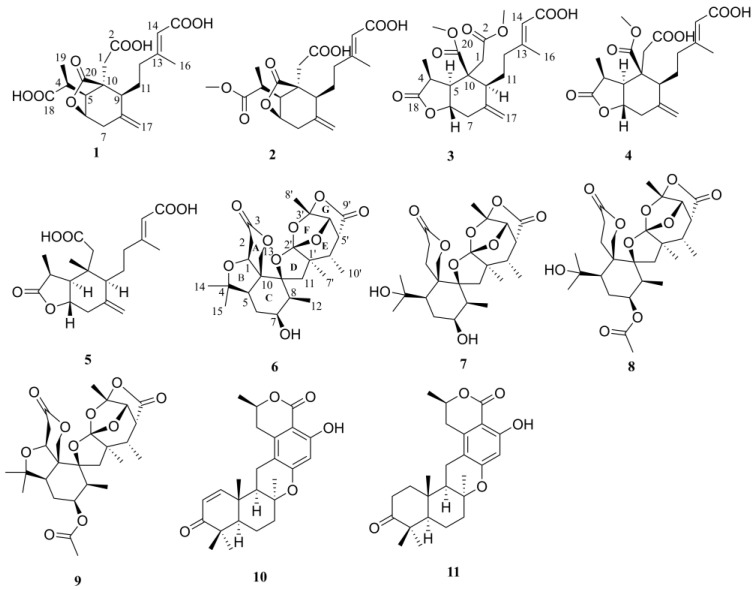
Chemical structures of **1**–**11**.

As part of our ongoing efforts to explore structurally novel and biologically active metabolites from marine-derived fungi [18,19,20,21], six new *seco*-terpenoids, talaroterpenoids A–F (**1**–**3** and **6**–**8**), together with five known compounds, 5-(4-carboxy-3-methyl-3-buten-1-yl) octahydro-4-(methoxycarbonyl)-3-methyl-6-methylene-2-oxo-4-benzofuranacetic acid (**4**) [22], penioxalicin (**5**) [22], fusariumin A (**9**) [23], verruculide A (**10**) [24] and pentacecilide A (**11**) [25] (Figure 1), were isolated from the marine-derived fungus *Talaromyces aurantiacus* (CGXWFNi1–24). Compounds **1** and **2** represent unusual 3-*nor*-labdane-type diterpenes with a 6,20-*γ*-lactone-bridged scaffold. Herein, we report the isolation, structure elucidation, possible biosynthetic pathway, and biological activities of these compounds.

## 2. Results and Discussion

**Figure 2:**
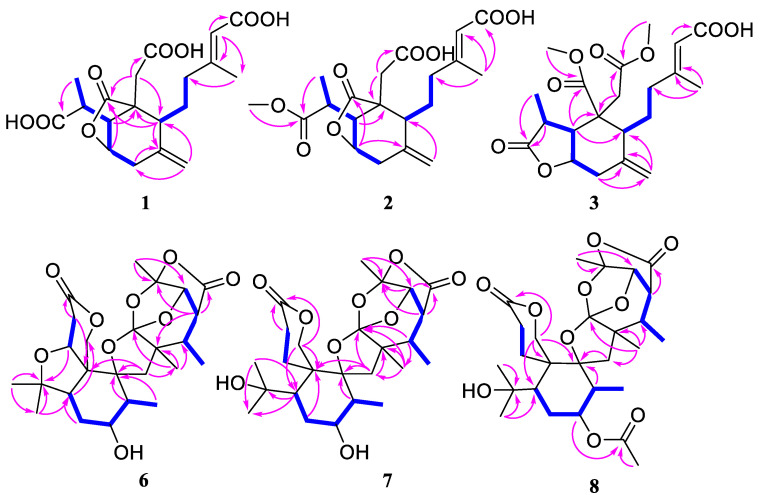
Key 1H-1H COSY (bold) and HMBC (arrows) correlations of talaroterpenoids A–F (**1**–**3** and **6**–**8**).

Talaroterpenoid A (**1**), isolated as a colorless oil, was assigned the molecular formula C_19_H_24_O_8_ by HRESIMS data at *m*/*z* 403.1357 [M + Na]^+^ (calcd for C_19_H_24_O_8_Na^+^, 403.1363), indicating 8 degrees of unsaturation. Analysis of its ^13^C NMR data (Table 1) revealed the presence of two methyls (δ_C_ 14.0 and 19.2) and four carbonyl carbons (δ_C_ 169.6, 174.0, 178.2, and 178.9). The ^1^H NMR data (Table 1) presented characteristic signals related to two methyls (δ_H_ 1.47 and 2.39), four methylenes (δ_H_ 2.31/1.91, 2.48/2.26, 2.84/2.70, and 3.56/3.09), four methines (δ_H_ 3.28, 3.47, 3.73, and 5.18), and three olefinic protons (δ_H_ 5.05, 5.07, and 6.16). The COSY spectrum revealed two distinct spin systems corresponding to H-19/H-4/H-5/H-6/H-7 and H-9/H-11/H-12 (Figure 2). Analysis of HMBC correlations of H-5 and H-9/C-10 and H-7 and H-9/C-8 indicated the presence of a cyclohexane ring. Furthermore, HMBC correlations of H_3_-19/C-18 demonstrated that a carboxyl group (C-18) was attached to C-4. The α,β-unsaturated acid residue was found to be adjacent to C-12, supported by HMBC correlations of H-14/C-13, C-15, and C-16, as well as H_3_-16/C-12. The connection of C-17 to C-8 was established through HMBC correlations of H_2_-17/C-7 and C-9. The carboxymethyl group was identified as being attached to C-10, based on HMBC correlations of H_2_-1/C-2, C-9, and C-20. Finally, the unusual 6,20-lactone-bridged moiety was established by the HMBC correlation of H-1 and H-6/C-20. Thus, the planar structure of **1** was established.

**Figure 3:**
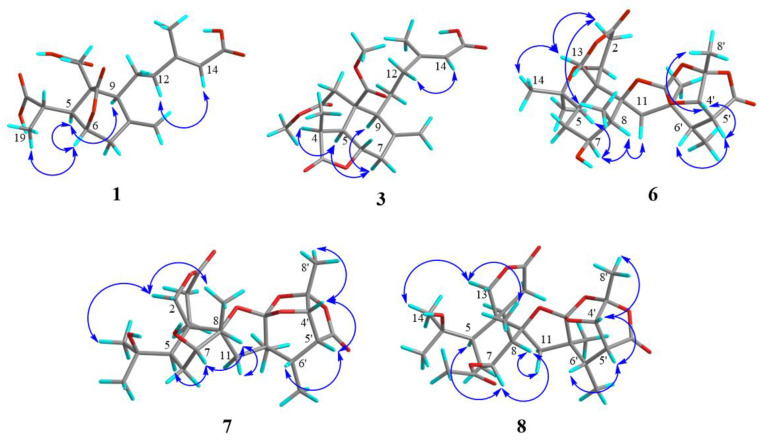
Key NOESY correlations of talaroterpenoids A, C, and D–F (**1**, **3**, and **6**–**8**).

**Figure 4:**
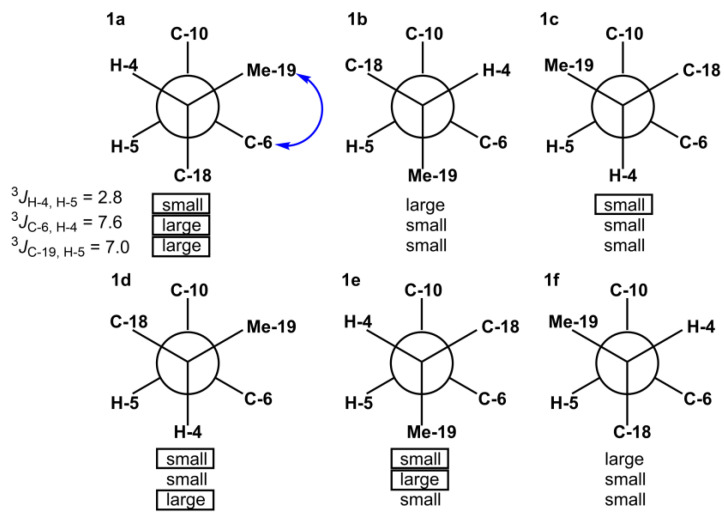
Newman projections for C-4/C-5 of **1**. All possible relative conformations are shown: 4S*,5S* (**1a**–**1c**) and 4R*, 5S* (**1d**–**1f**). The observed NOESY correlation is presented as an arrowed line.

**Figure 5:**
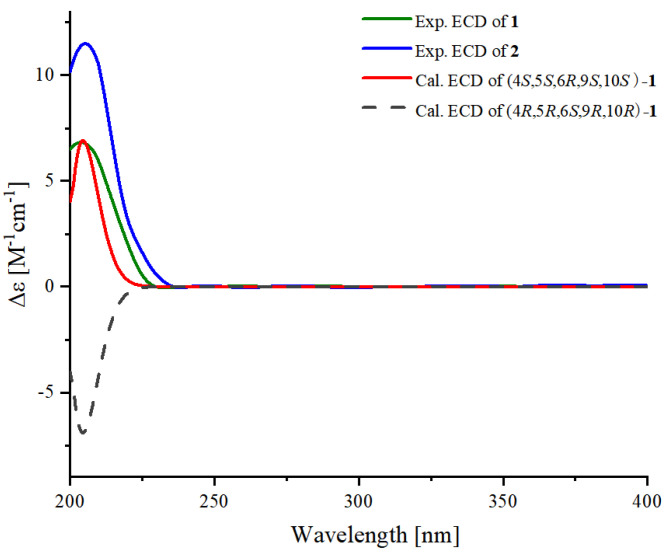
Experimental ECD spectra for compounds **1** and **2** in MeOH and calculated ECD spectra for (4*S*, 5*S*, 6*R*, 9*S*, 10*S*)-**1** and (4*R*, 5*R*, 6*S*, 9*SR*, 10*R*)-**1**.

The relative configuration of the cyclohexane ring moiety in **1** was established by analysis of the NOESY correlations (Figure 3) and coupling constants. The NOESY correlation of H-9 with H-5 suggested that they were cofacial and in an axial arrangement with an arbitrarily assigned α-orientation. Furthermore, NOESY correlations of H-5 with H-6 suggested that H-6 was also in the α-orientation. The small coupling constant of ^3^*J*_H-5,H-6_ (broad) confirmed that H-6 was in an equatorial arrangement and oriented *cis* to H-5. The orientation of the carboxymethyl group must be α, *cis*-to H-6, because the alternative highly strained “in–out” *trans* intrabridged structure is incompatible with a 6,20-γ-lactone-bridged skeleton. The geometry of the double bond at C-13 in the side chain was assigned *E* because of the NOE correlation of H_2_-12/H-14. The relative configurations of the C-4 and C-5 were determined by the analysis of *J*_H,H,_ and *J*_C,H_ coupling constants [26] and NOESY correlations (Figure 4). Carbon–proton coupling constants (^2,3^*J*_C,H_) were measured by a *J*-resolved HMBC experiment. A small coupling constant between H-4 and H-5 (2.8 Hz) suggested that H-4 and H-5 were in the gauche orientation [27]. On the other hand, a strong NOESY correlation between H-6/H-19 indicated a gauche orientation. Finally, large ^3^*J*_C-6,H-4_ (7.6 Hz) and ^3^*J*_C-19,H-5_ (7.0 Hz) indicated that C-6 and H-4 and C-19 and H-5 were in the *anti*-orientation, respectively. Thus, the relative configuration of **1** was determined as 4S*, 5S*, 6R*, 9S*, 10S*, and the ∆^13^ double bond was determined as *E*-configuration. To further ascertain the absolute configuration of **1**, the electronic circular dichroism (ECD) calculation was employed. ECD calculations for (4*S*, 5*S*, 6*R*, 9*S*, 10*S*)-**1** and (4*R*, 5*R*, 6*S*, 9*R*, 10*R*)-**1** were conducted using the time-dependent density functional theory (TDDFT) at the B3LYP-D3(BJ)/6–31G* level. The calculated ECD spectra for (4*S*, 5*S*, 6*R*, 9*S*, 10*S*)-**1** matched with the experimental curve well (Figure 5), which indicated that the absolute configuration of **1** was 4*S*, 5*S*, 6*R*, 9*S*, and 10*S*.

Talaroterpenoid B (**2**) was obtained as a colorless oil, and its molecular formula was assigned as C_20_H_26_O_8_ by HRESIMS, requiring 8 degrees of unsaturation. The NMR data of **2** (Table 1) were similar to those of **1** (^1^H and ^13^C NMR data in DMSO-*d*_6_), with the significant difference being the presence of a methyl ester group (δ_C_ 52.2, δ_H_ 3.62) in **2**. The HMBC correlations (Figure 2) of O-Me/C-18 suggested that the structure of **2** was the methylated derivative of **1**. Detailed analysis of the 2D NMR data supported the proposed planar structure for **2**. Furthermore, the close similarity of ^1^H and ^13^C NMR shifts as reported in Table S1, as well as the *J* values between **2** and **1**, indicated that they shared the same relative configuration. Compounds **2** and **1** showed similar ECD spectra (Figure 5), indicating that they share the same absolute configuration, 4*S*, 5*S*, 6*R*, 9*S*, and 10*S*.

**Figure 6:**
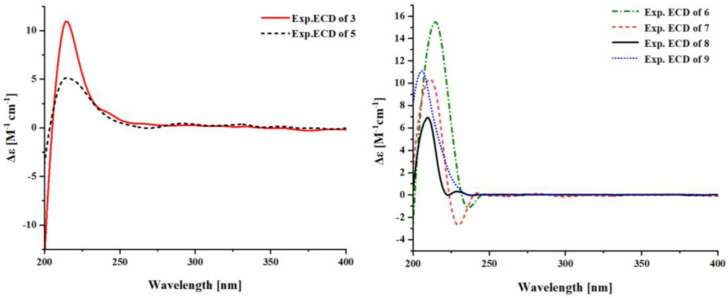
Experimental ECD spectra of compounds **3**, **5**, and **6**–**9** (in MeOH).

Talaroterpenoid C (**3**) was obtained as a white powder. Its molecular formula was determined as C_21_H_28_O_8_ on the basis of HRESIMS data at *m*/*z* 409.1868 [M + H]^+^ (calcd for C_21_H_29_O_8_, 409.1862), which required 8 degrees of unsaturation. The NMR data resembled those of **5** [13]. The observation of two additional methoxyl groups (*δ*_H/C_ 3.66/52.2 and 3.71/52.3), coupled with the oxidation of one of the methyl groups in **5** to an ester carbonyl group (*δ*_C_ 171.7), collectively indicated that **3** was an oxidized derivative of **5**. Furthermore, HMBC correlations of H_3_-21 and H-5/C-20, H_2_-1 and H_3_-22/C-2, as well as H_2_-1 and H-5/C-10, indicated that the methyl ester group (C-20) and a carboxymethyl group (C-1) were attached to C-10. Based on the NMR data of **3** and **5** as reported in Table S2, along with the NOESY correlations of **3**, the relative configuration of **3** was determined. Furthermore, the CD spectra of **3** and **5** showed a positive CE around 213 nm in the lactone absorbance region (Figure 6), indicating that the absolute configuration of **3** would be 4*S*, 5*S*, 6*S*, 9*S*, and 10*S*.

**Figure 7:**
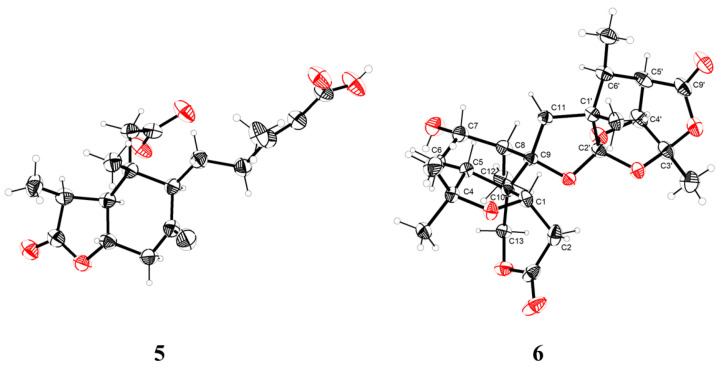
X-ray crystallographic analysis of **5** and **6**.

Talaroterpenoid D (**6**) was obtained as a white solid. The molecular formula was determined by HRESIMS analysis to be C_25_H_34_O_9_, with nine degrees of unsaturation. Inspection of the NMR and HRESIMS data of **6** revealed the presence of the orthoester-containing meroterpenoid skeleton. A detailed analysis of its NMR spectra revealed strong similarities to those of fusariumin A (**9**) [23] as reported in Table S3. However, the acetyl (δ_H_ 2.01; δ_C_ 21.2, 170.2 in **9**) was absent in **6**. Extensive analysis of 2D NMR revealed the planar structure of **6** as described in Figure 1. The relative configuration of talaroterpenoid D (**6**) was determined by comparison of its ^1^H and ^13^C NMR data with those of **9**. The absolute configuration of **6** (1*S,* 5*R*, 7*S*, 8*S*, 9*S*, 10*S*, 1′*R*, 2′*S*, 3′*S*, 4′*S*, 5′*R*, 6′*S*) was determined (Figure 4) by X-ray diffraction analysis using Cu Kα radiation with a Flack parameter of 0.06 (9) (Figure 7). Talaroterpenoid D (**6**) was a polycyclic spiromeroterpenoid whose structure possessed a congested heptacyclic skeleton with a 6/5/6/5/6/5/5 system.

Talaroterpenoid E (**7**) was obtained as a white amorphous powder, possessing a molecular formula of C_25_H_36_O_9_ with 8 degrees of unsaturation. The ^1^H and ^13^C NMR spectra (Table 2) of **7** were similar to those of **6,** with the main difference at the fission of the tetrahydrofuran ring of **7** at C-1 (δ_H_ 4.16, δ_C_ 74.7 in **6**; δ_H_ 1.80, 2.70, δ_C_ 26.0 in **7**). Extensive analysis of 2D NMR revealed the planar structure of **7** as described (Figure 2). The relative structure of **7** was determined by comparing its NMR data with those of **6** and further substantiated by a detailed analysis of NOESY data (Figure 3). Key NOESY correlations of H-5/H-7, H-7/H-8, H-8/H-11β, H_2_-12/H-13, and H-13/H_3_-14 determined the relative configuration of the A/C ring (Figure 1). Furthermore, the NOESY correlations of H-4′/H-5′, H-5′/H-6′, H-4′/H_3_-8′, and H_3_-7′/H_3_-10′ indicated the relative configuration of the E/F ring. Compounds **7** and **6** showed quite similar ECD spectra (Figure 6), with a positive cotton effect (CE) at 210 nm, indicating that they share the same absolute configuration. The absolute configuration of **7** was established as 5*R*, 7*S*, 8*S*, 9*S*, 10*S*, 1′*R*, 2′*S*, 3′*S*, 4′*S*, 5′*R*, 6′*S.*

Talaroterpenoid F (**8**) was isolated as a colorless amorphous solid, and its molecular formula was determined as C_27_H_38_O_10_ (nine degrees of unsaturation) on the basis of HRESIMS. The ^1^H and ^13^C NMR spectra (Table 2) of **8** were very similar to those of **7**, except that **8** has one more acetyl group [C-16 (δ_C_ 169.9), CH_3_-17 (δ_H_ 1.99, δ_C_ 20.9) than **7**. The planar structure of **8** was mainly elucidated by 2D NMR. The absolute configuration of **8** was the same as that of **7**, based on their comparable NMR data and CD spectra.

**Scheme 1:**
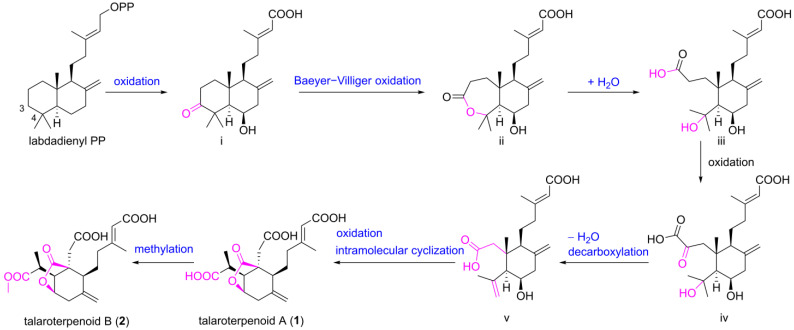
Hypothetical biosynthetic pathway of talaroterpenoids A and B (**1** and **2**).

Structurally, talaroterpenoids A–C (**1**–**3**) are 3-*nor*-2,3-*seco*-labdane type diterpenes with four carbonyl groups. The 3-*nor*-labdane-type compounds are rare in nature. There are only four compounds with the 3-*nor*-2,3-*seco*-labdane skeleton having been reported from nature sources, including penioxalicin [17], isolated from the fungus *Penicillium oxalicum* TW01–1; penicichrysogene A [28], isolated from the endophytic fungus *P*. *chrysogenum* MT-12; paecilomycine A [29], isolated from the insect pathogenic fungus *Paecilomyces* sp. ACCC 37762; and 5-(4-carboxy-3-methyl-3-buten-1-yl)octahydro-4-(methoxycarbonyl)-3-methyl-6-methylene-2-oxo-4-benzofuranacetic acid [22], isolated from the marine-derived fungus *Talaromyces* sp. PSU-MF07. Talaroterpenoids D–F (**6**–**8**) and fusariumin A (**9**) are orthoester groups containing 3,4-*seco*-meroterpenoid featuring highly oxygenated seven/six-membered rings, which bear three quaternary spirocarbons. Up to now, only 10 natural products containing an orthoester group have been reported from fungi. They are amaurone E [30], asnovolins F and G [31], novofumigatonin [32], novofumigatonol, fumigatonoid C [33], (+)-peniorthoesters A and B, and (–)-peniorthoesters A and B [34]. Among these compounds, novofumigatonin was investigated for its biosynthetic pathway, and the key enzymes for the orthoester formation have been identified by a series of gene-deletion experiments [33]. Particularly, **1** and **2** featured an unusual 6,20-*γ*-lactone-bridged scaffold. A plausible biogenetic pathway of **1** and **2** was proposed as shown in Scheme 1. Talaroterpenoids A (**1**) and B (**2**) may be derived from the precursor of labdadienyl PP [17]. Oxidation of anticopalic acid could initiate an anticopalic acid analogue i, and a further Baeyer–Villiger oxidation on i could give intermediate ii. The hydrolysis ring opening of ii at C-3 delivers intermediate iii. Then, oxidation at C-2 could produce intermediate ⅳ, and dehydration and decarboxylation may afford intermediate ⅴ. subsequently, oxidation, lactonization, and methylation could lead to the formation of talaroterpenoids A and B (**1** and **2**) with *γ*-lactone-bridged 3-*nor*-2,3-*seco*-diterpenes.

Recent investigations have revealed that neuroinflammation, partially attributed to deregulated microglial activation, contributes to the onset and progression of neurodegenerative conditions, notably AD and depressive-like manifestations [35]. In the central nervous system (CNS), activated microglial cells are capable of generating nitric oxide (NO). An excessive accumulation of NO within the CNS, stemming from the induction of nitric oxide synthase (iNOS), can precipitate unchecked neuroinflammatory responses [36]. Hence, in order to identify potent anti-neuroinflammatory agents from the isolated compounds, the cytotoxicity of **1**–**11** was evaluated in BV-2 cells. All compounds did not show toxicity at the concentrations of 10 and 50 μM (Figure S1). We then assessed their capacity to inhibit NO production employing an in vitro neuroinflammation model, wherein BV-2 cells were stimulated with LPS. The results showed that at a concentration of 50 μM, only compounds **10** and **11** exhibited an inhibition rate greater than 80% (Figure S2). Subsequently, the IC_50_ values of these two compounds were determined to be 11.47 ± 0.39 and 11.32 ± 0.72 μM, respectively, surpassing the efficacy of the positive control minocycline (IC_50_ = 22.9 ± 0.8 μM). This is the first report of the anti-neuroinflammatory activity of compounds **10** and **11**.

The antimicrobial activities of compounds **1**–**11** were also evaluated in vitro against four plant pathogenic fungi (*Alternaria alternata*, *Pestalotiopsis theae* Steyaert, *Thielaviopsis paradoxa*, and *Colletotrichum capsici*) and six bacteria (*Staphylococcus aureus*, *Escherichia coli*, *Enterococcus faecalis*, *Shigella dysentery*, *Vibrio parahemolyticus*, and *Bacillus subtilis*). Compound **3** exhibited antifungal activities against *A. alternata* and *P. theae* Steyaert, with the MIC (minimum inhibitory concentration) value of 50 μg/mL. Compound **5** exhibited an effect on *A. alternata* with the MIC value of 50 μg/mL. All compounds (**1**–**11**) do not exhibit antibacterial activity at a concentration of 100 μg/mL. Carbendazim was employed as the positive control, exhibiting a MIC value of 25 μg/mL against *A. alternata*, whereas *P. theae* Steyaert demonstrated a MIC value of 50 μg/mL.

## 3. Materials and Methods

### 3.1. General Experimental Procedures

Optical rotations were analyzed utilizing a JASCO P-2000 polarimeter (Jasco, Tokyo, Japan). UV spectral measurements were conducted on a Cary 60 spectrophotometer (Jasco, Tokyo, Japan). ECD spectra were acquired using a JASCO J-1500 spectropolarimeter (Jasco, Tokyo, Japan). IR spectra were collected using KBr disks on a PerkinElmer Spectrum Two FT-IR spectrometer (Bruker, Ettlingen, Germany). NMR spectra were recorded on a Bruker AV-400 NMR spectrometer (Bruker, Bremen, Germany), with chemical shifts reported in *δ* values relative to the residual solvent peak. High-resolution ESI-MS (HRESIMS) data were collected on an Agilent 6545 Q-TOF LC-MS spectrometer (Agilent Technologies, Waldbronn, Germany). The analytical instruments employed for the purified compounds and the isolation procedure materials were consistent with those previously documented [37].

### 3.2. Biological Material

The fungal strain CGXWFNi1–24 was isolated from a sea sediment sample that was collected by dredge from the South China Sea (latitude 108.2519 E, longitude 21.5074 N; Figure S47) at a depth of −6 m. The fungus was identified according to a molecular biological protocol by DNA amplification and sequencing of the ITS region. Its 558 base pair ITS sequence (TTGATATGCTTAAGTTCAGCGGGTAACTCCTACCTGATCCGAG GTCAACCGTGGTAAAACTGTGGTGGTGACCAACCTCCGCCGATCCGTCCCGAGCGATGACAAAGCCCCATACGCTCGAGGACCAGACGGACGTCGCCGCTGCCTTTCGGACAGGTCCCCGGGGGGACCACGCCCAACACACAAGCCGGGCTTGAGGGCAGAAATGACGCTCGGACAGGCATGCCCCCCGGAATGCCAGGGGGCGCAATGTGCGTTCAAAGATTCGATGATTCACGGAATTCTGCAATTCACATTACTTATCGCATTTCGCTGCGTTCTTCATCGATGCCGGAACCAAGAGATCCATTGTTGAAAGTTTTGACAATTTTCATTCGACTCAGACAGCCCATCTTCATCAGGGTTCACAGAGCGCTTCGGCGGGCGCGGGCCCGGGGACGGACGTCCCCCGGCGACCGGGTGGCCCCGGTGGGCCCGCCAAAGCAACAGGTAGAGAGAGACACGGGGGGGGAGGTTGGGCCGCGAGGGCCCGCACTCGGTAATGATCCTTCCGCAGG) had 99.64% sequence identity to that of *Talaromyces aurantiacus* (MH857871). The strain was deposited in the State Key Laboratory for Chemistry and Molecular Engineering of Medicinal Resources, Guangxi Normal University, Guilin, P.R. China, with the GenBank (NCBI) access number OR016169.

### 3.3. Extraction and Isolation

The strain was inoculated into solid medium in 100 Erlenmeyer flasks (1 L), each flask containing 80 g of rice, 0.9 g of sea salt, and 100 mL of water. The fungus was fermented at 28 °C for 4 weeks before harvesting.

A total of 100 flasks undergoing fermentation were extracted three times with an equal volume of ethyl acetate (EtOAc). The organic solvent was then removed under vacuum, yielding an extract of 65.1 g. The extract was subsequently processed through vacuum-liquid chromatography (VLC) using a silica gel column. A gradient elution of EtOAc-petroleum ether (10:90 to 100:0, *v*/*v*) was employed to fractionate the extract into four subfractions (Fr.1–Fr.4). Fr.3 produced precipitation Fr.3.0 (353.0 mg). Supernatant was separated into 23 subfractions (Fr.3.1–Fr.3.23) in an ODS column (MeOH-H_2_O, 30:70–100:0). Fr.3.8 (234.0 mg) underwent purification by HPLC (MeCN-H_2_O, 30:70) to yield **4** (26.8 mg). Similarly, Fr.3.10 (116.1 mg) was further separated by HPLC (MeCN-H_2_O, 33:67) to yield **1** (63.3 mg) and **2** (7.8 mg). Fr.3.12 (184.0 mg) was subjected to additional separation by HPLC (MeCN-H_2_O, 35:65) to yield **3** (9.3 mg) and **5** (31.7 mg). Fr.3.0 further subjected to HPLC (MeCN-H_2_O, 30:70–80:20, 35 min) to yield **6** (10.0 mg), **7** (143.9 mg), **8** (50.3 mg), and **9** (37.3 mg). Fr.2 (10.0 g) was separated into 9 subfractions (Fr.2.1–Fr.2.9) in an ODS column (EtOAc-petroleum, 30:70–100:0). Fr.2.3 (174.8 mg) was fractionated by an ODS column (MeOH-H_2_O, 10:90–100:0). Subsequent purification of Fr.2.3.5 through ODS chromatography yielded **10** (63.0 mg) and **11** (42.3 mg).

#### 3.3.1. *Talaroterpenoid A* (**1**)

Colorless oil; ${\left[\alpha \right]}_{\;D}^{25}$: + 9.5 (*c* 0.1, MeOH); UV (MeOH) *λ*_max_ (log *ε*): 211 (4.19); IR *ν*_max_ 3419, 2927, 1709, 1644, 1392, and 1199 cm^−1^. CD (MeOH) *λ*_max_ (Δ*ε*): 204 (+6.82); ^1^H NMR and ^13^C NMR data, see Table 1; HRESIMS *m*/*z* 403.1357 [M + Na]^+^ (calcd for C_19_H_24_O_8_Na^+^, 403.1363).

#### 3.3.2. *Talaroterpenoid B* (**2**)

Colorless oil; ${\left[\alpha \right]}_{\;D}^{25}$: + 7.9 (*c* 0.1, MeOH); UV (MeOH) *λ*_max_ (log *ε*): 215 (4.23); IR *ν*_max_ 3419, 2926, 2854, 1771, 1730, 1396, and 1241 cm^−1^. CD (MeOH) *λ*_max_ (Δ*ε*): 204 (+11.46); ^1^H NMR and ^13^C NMR data, see Table 1; HRESIMS *m*/*z* 417.1521 [M + Na]^+^ (calcd for C_20_H_26_O_8_Na^+^, 417.1520).

#### 3.3.3. *Talaroterpenoid C* (**3**)

Colorless oil; ${\left[\alpha \right]}_{\;D}^{25}$: + 21.9 (*c* 0.1, MeOH); UV (MeOH) *λ*_max_ (log *ε*): 202 (3.71), 216 (3.64); IR *ν*_max_ 3422, 2966, 2856, 1730, 1444, and 1212 cm^−1^. CD (MeOH) *λ*_max_ (Δ*ε*): 216 (+4.98); ^1^H NMR and ^13^C NMR data, see Table 1; HRESIMS *m*/*z* 409.1868 [M + H]^+^ (calcd for C_21_H_29_O_8_^+^, 409.1857), 431.1683 [M + Na]^+^ (calcd for C_21_H_28_O_8_Na^+^, 431.1677).

#### 3.3.4. *Talaroterpenoid D* (**6**)

White amorphous powder; ${\left[\alpha \right]}_{\;D}^{25}$: + 37.1 (*c* 0.1, MeOH); IR *ν*_max_ 3433, 2942, 1714, 1621, 1239, and 1053 cm^−1^. CD (MeOH) *λ*_max_ (Δ*ε*): 214 (+ 15.38); ^1^H NMR and ^13^C NMR data, see Table 2; HRESIMS *m*/*z* 479.2277 [M + H]^+^ (calcd for C_25_H_34_O_9_^+^, 479.2281), 431.1683 [M + Na]^+^ (calcd for C_21_H_28_O_8_Na^+^, 501.2092).

#### 3.3.5. *Talaroterpenoid E* (**7**)

White amorphous powder; ${\left[\alpha \right]}_{\;D}^{25}$: + 17.9 (*c* 0.1, MeOH); IR *ν*_max_ 3436, 2922, 1734, 1631, 1242, and 1068 cm^−1^. CD (MeOH) *λ*_max_ (Δ*ε*): 212 (+ 10.33); ^1^H NMR and ^13^C NMR data, see Table 2; HRESIMS *m*/*z* 503.2249 [M + Na]^+^ (calcd for C_25_H_36_O_9_Na^+^, 503.2257).

#### 3.3.6. *Talaroterpenoid F* (**8**)

White amorphous powder; ${\left[\alpha \right]}_{\;D}^{25}$: + 20.6 (*c* 0.1, MeOH); IR *ν*_max_ 3427, 2924, 1751, 1635, 1245, and 1027 cm^−1^. CD (MeOH) *λ*_max_ (Δ*ε*): 210 (+ 6.92); ^1^H NMR and ^13^C NMR data, see Table 2; HRESIMS *m*/*z* 523.2531[M + H]^+^ (calcd for C_27_H_39_O_10_^+^, 523.2543), 545.2375 [M + Na]^+^ (calcd for C_27_H_38_O_10_Na^+^, 545.2363).

### 3.4. Computational Section

Conformational analyses were performed utilizing the “systematic” approach incorporated into Sybyl-X 2.0, which made use of the MMFF94S force field and an energy cutoff threshold set at 5 kcal/mol. This analysis uncovered nine conformers possessing the lowest energy states. Following this, geometry optimizations and frequency analyses were executed at the B3LYP-D3(BJ)/6–31G* level within the CPCM methanol solvation model, all facilitated by ORCA5.0.1 [38]. Each conformer utilized in subsequent property calculations was verified as a stable point on the potential energy surface (PES), confirmed by the absence of imaginary frequencies. Excitation energies, oscillator strengths, and rotational strengths (velocity) for the first 60 excited states were computed using the TD-DFT method at the PBE0/def2-TZVP level in CPCM methanol, again employing ORCA5.0.1. The ECD spectra were generated by applying overlapping Gaussian functions, with a half-bandwidth corresponding to 1/e of the peak height (sigma = 0.30 for all conformers) [39]. Gibbs free energies for the conformers were determined by applying thermal correction at the B3LYP-D3(BJ)/6–31G* level, and electronic energies were evaluated at the wB97M-V/def2-TZVP level in CPCM methanol using ORCA5.0.1. The final spectra were obtained by averaging the simulated spectra of the conformers, weighted according to the Boltzmann distribution theory and their relative Gibbs free energy differences (∆G).

### 3.5. X-ray Crystallographic Analyses

The clear, colorless crystals of compounds **5** and **6** were obtained in MeOH by slow evaporation. X-ray crystallographic data were collected utilizing a Rigaku Oxford Diffraction Supernova Dual Source instrument (Agilent SuperNova, Santa Clara, CA, America), specifically the Cu at Zero configuration, which was equipped with an AtlasS2 CCD detector. Measurements for compounds **5** and **6** were conducted at a temperature of 297.00(10) K, employing Cu Kα radiation with a wavelength of 1.54184 Å. The structures of these compounds were determined through direct methods facilitated by the Olex2 software package (Olex2-1.5) [40], followed by anisotropic refinement using SHELXL-2018 [41]. The refinement process was carried out using a full-matrix least-squares approach based on F2. Crystallographic data (Table S1, Supporting Information) have been deposited at the Cambridge Crystallographic Data Center under deposition numbers CCDC 2264255 (**5**) and 2385763 (**6**). These data can be obtained free of charge via the internet at www.ccdc.cam.ac.uk (accessed on 20 May 2023 for **5** and 23 September 2024 for **6**).

### 3.6. Anti-Neuroinflammatory Activity

Cell viability assessment was conducted utilizing a cell counting kit-8 (CCK8, sourced from Beyotime Biotechnology, Shanghai, China) [42]. In brief, BV-2 cells were plated at a density of 4 × 10^4^ cells per well in a 96-well culture plate, each well containing 100 μL of medium, and incubated for 24 h. Subsequently, the medium in both the normal control and lipopolysaccharide (LPS) groups was exchanged with fresh medium, whereas the sample groups were exposed to compounds at concentrations of 10 and 50 μM. After 1 h, fresh medium was introduced to the normal control group, while the remaining groups received medium supplemented with 200 ng/mL of LPS. To evaluate the inherent cytotoxicity of the compounds, BV-2 cells were also treated with the compounds in the absence of LPS. Following a 24 h incubation, 10 μL of CCK8 solution was administered to each group, followed by an additional 30 min incubation at 37 °C. Absorbance at 450 nm (A450) was then quantified using a microplate reader (BioTek, Winooski, VT, USA).

The capacity of compounds **10** and **11** to inhibit NO production was assessed utilizing Griess reagents [42]. Briefly, a suspension of 4 × 10^4^ BV-2 cells in 100 μL medium was dispensed into each well of a 96-well cell culture plate. Following a 24 h incubation period, the medium in both the normal control and LPS-treated groups was exchanged with fresh medium, whereas the remaining groups received medium supplemented with varying concentrations (10 and 50 μM for inhibition rate, and 1.56, 3.12, 6.25, 12.5, 25, 50, and 100 μM for IC_50_) of the compounds or the positive control (minocycline). Subsequent to a 1 h incubation at 37 °C, fresh medium was added to the normal control group, whereas the other groups were exposed to 200 ng/mL of LPS. After an additional 24 h of incubation, 70 μL of the culture medium was transferred to a fresh 96-well plate, and 80 μL of Griess reagents were added to each well. Following a 10 min incubation at 37 °C, A_540_ was determined using a microplate reader.

### 3.7. Antimicrobial Activities

The antimicrobial activities of compounds **1**–**11** were assessed using the serial-dilution method, in accordance with previously reported literature [43,44,45]. Briefly, the test compounds were dissolved in DMSO and subsequently introduced into the nutrient media, comprising beef extract broth for bacterial cultures and potato dextrose broth (PDB) for fungal cultures, to achieve the requisite concentrations spanning 6.25 to 100.0 μg/mL. Inoculation of bacterial and fungal cultures, each with a density of 10^6^ cells (or spores) per milliliter, was then carried out in the respective media. The bacterial cultures were incubated at 37 °C for 24 h, whereas the fungal cultures were maintained at 28 °C for 48–72 h. The growth of the microorganisms was monitored by assessing the turbidity of the nutrient media. The MIC for each compound was defined as the lowest concentration that resulted in the complete inhibition of visible microbial growth. As positive controls for bacterial and plant pathogenic fungi, ciprofloxacin and carbendazim were utilized, respectively.

## 4. Conclusions

Six new highly oxidized 3,4-*seco*-terpenoids, talaroterpenoids A–F (**1**–**6**), were isolated from the marine-derived fungus *T. aurantiacus*. Compounds **1** and **2** were labdane type diterpenes featuring an unusual 6,20-γ-lactone-bridged scaffold. Talaroterpenoids D–F (**6**–**8**) possesses an orthoester group, which was rare reported from fungi. Verruculide A (**10**) and pentacecilide A (**11**) showed significant NO production inhibition in lipopolysaccharide (LPS)-induced BV-2 cells, while talaroterpenoid C (**3**) exhibited moderate antifungal activities against the plant pathogenic fungi *A. alternata* and *P. theae Steyaert*, with the MIC value of 50 μg/mL.

## Figures and Tables

**Table 1:** The NMR data (^1^H 400 MHz, ^13^C 100 MHz) for talaroterpenoids A–C (**1**–**3**).

No.	1 *^a^*		2 *^b^*		3 *^c^*	
	*δ*_C_	*δ*_H_ (*J* in Hz)	*δ*_C_	*δ*_H_ (*J* in Hz)	*δ*_C_	*δ*_H_ (*J* in Hz)
1	34.0	3.56, d (17.6) 3.09, d (17.6)	32.3	2.77, overlapped 2.48, overlapped	37.4	3.12, d (15.0) 2.66, d (15.0)
2	174.0		171.7		170.9	
4	39.2	3.47, qd (7.2, 2.8)	37.5	2.77, overlapped	37.8	2.86, q (7.4)
5	52.2	3.73, d (2.8)	50.6	2.89, d (3.0)	51.7	2.46, dd (11.4, 7.4)
6	78.2	5.18, dd (3.4, 2.4)	76.6	4.74, t (3.0)	77.3	4.93, td (11.4, 4.8)
7	40.1	2.84, dd (15.2, 3.4) 2.70, dd (15.2, 2.4)	39.7	2.64, overlapped 2.54, overlapped	42.1	2.23, t (11.8) 3.03, dd (11.8, 4.8)
8	143.8		142.4		141.0	
9	49.7	3.28, br d (10.0)	48.0	2.67, overlapped	48.0	2.35, m
10	50.9		48.8		50.7	
11	26.5	2.31, m 1.91, m	24.8	1.70, m 1.40, m	23.5	1.75, m
						1.42, m
12	40.2	2.48, m 2.26, m	38.8	2.25, m 1.96, m	39.8	2.35, m
						2.07, m
13	158.6		158.4		162.1	
14	118.5	6.16, d (1.3)	116.5	5.54, s	115.4	5.67, s
15	169.6		167.5		171.2	
16	19.2	2.39, d (1.3)	18.3	2.05, s	19.4	2.18, s
17	116.1	5.07, s	115.3	5.03, s	114.0	5.18, s
		5.05, s		4.88, s		4.83, s
18	178.2		174.9		178.5	
19	14.0	1.47, d (7.2)	13.0	0.96, d (7.2)	10.1	1.06, d (7.4)
20	178.9		177.2		171.7	
21			52.2	3.62, s	52.2	3.66, s
22					52.3	3.71, s

*^a^* Recorded in pyridine-*d*_5_. *^b^* Recorded in DMSO-*d*_6_. *^c^* Recorded in CDCl_3_.

**Table 2:** The NMR data (400 MHz for ^1^H and 100 MHz for ^13^C in DMSO-*d*_6_) of talaroterpenoids D–F (**6**–**8**).

No.	6		7		8	
	*δ*_C_	*δ*_H,_ (*J* in Hz)	*δ*_C_	*δ*_H,_ (*J* in Hz)	*δ*_C_	*δ*_H,_ (*J* in Hz)
1	74.7	4.16, d (4.2)	26.0	2.70, m	25.9	2.70, m
				1.80, m		1.82, m
2	28.8	2.56, m 2.68, dd (15.8, 4.2)	29.1	2.36, m	29.1	2.39, m
				2.37, m		2.33, m
3	170.0		173.3		173.3	
4	79.6		73.1		73.1	
5	50.5	1.57, d (8.2)	49.1	1.46, br d (12.4)	48.9	1.43, br d (12.8)
6	25.2	1.52, m	25.8	1.40, m 1.46, m	25.5	1.69, m 1.52, m
7	69.2	3.69, m	67.1	3.70, dt (11.2, 5.2)	71.7	4.81, dt (12.4, 5.0)
8	49.1	2.19, m	46.6	2.54, s	43.2	2.59, m
9	86.9		92.2		91.4	
10	49.5		44.7		44.8	
11	48.8	1.86, d (14.2) 2.41, d (14.2)	49.7	1.92, d (14.0) 2.03, d (14.0)	49.7	2.12, d (14.0) 1.95, d (14.0)
12	10.3	0.87, d (7.4)	10.2	0.89, d (7.4)	10.3	0.93, d (7.4)
13	65.5	4.45, d (13.0) 4.52, d (13.0)	66.1	4.26, d (12.2) 4.47, d (12.2)	66.5	4.53, d (12.2) 4.27, d (12.2)
14	21.9	0.98, s	34.3	1.19, s	34.1	1.19, s
15	30.9	1.18, s	25.8	1.14, s	25.9	1.14, s
16					169.9	
17					20.9	1.99, s
1′	46.8		47.0		47.0	
2′	127.8		128.0		128.0	
3′	107.3		107.3		107.3	
4′	78.2	5.04, d (8.4)	77.6	5.02, d (8.6)	77.6	5.02, d (8.4)
5′	41.3	2.83, dd (8.4, 6.2)	41.3	2.81, dd (8.6, 6.0)	41.2	2.83, dd (8.4, 6.0)
6′	38.9	2.07, m	40.4	2.02, m	39.1	2.02, m
7′	18.4	0.74, s	18.6	0.87, s	18.6	0.87, s
8′	21.9	1.62, s	22.1	1.63, s	22.0	1.62, s
9′	175.1		175.5		175.3	
10′	11.8	1.04, d (7.4)	11.7	1.05, d (7.0)	11.8	1.05, d (7.0)

## Data Availability

Data are contained within the article or Supplementary Material.

## Supplementary Materials

The following are available online at https://www.mdpi.com/article/10.3390/md22100475/s1, the NMR data of **1**–**3**, **5**, **6**, and **9**, anti-neuroinflammatory activity data, 1D (^1^H NMR, ^13^C NMR) spectra, 2D (COSY, HSQC, HMBC, and *J*-HMBC) spectra, and HRESIMS of new compounds **1**–**3** and **6**–**8**.
