# Supplementary material for: Talaroterpenoids A–F: Six New Seco-Terpenoids from the Marine-Derived Fungus Talaromyces aurantiacus
Source: Mar Drugs. 2024 Oct 18;22(10):475. doi: 10.3390/md22100475 (PMC11509394; doi:10.3390/md22100475)
Supplement: Supplementary file 1 [file marinedrugs-22-00475-s001.zip › marinedrugs-3258588-supplementary.pdf]

Supplementary Materials

Talaroterpenoids A–F: Six New Seco-Terpenoids from the Marine-Derived Fungus *Talaromyces aurantiacus*

Zi-Hong Peng [1,†], Hui Jia [1,†], Yan-Liang Luo [1], Li-Jun Zhang [1], Jia-Tong Zhou [1], Yuan-Han Xie [1], Li-Jun Wang [2], Jiang-Ke Qin [1], Jun Li [1], Guo-Hai Zhang [1,*], Rui-Yun Yang [1,*] and Wei-Feng Xu [1,*]

[1] State Key Laboratory for Chemistry and Molecular Engineering of Medicinal Resources, Collaborative Innovation Center for Guangxi Ethnic Medicine, School of Chemistry and Pharmaceutical Sciences, Guangxi Normal University, Guilin 541004, China; pengzihong000311@163.com (Z.-H.P.); jiahui18878391665@163.com (H.J.); lyl2010927@163.com (Y.-L.L.); ajun840618@mailbox.gxnu.edu.cn (L.-J.Z.); zjt990123@163.com (J.-T.Z.); xieyuanhan0330@163.com (Y.-H.X.); jiangke@sina.com (J.-K.Q.); lijun9593@gxnu.edu.cn (J.L.)

[2] School of Design, Guangxi Normal University, Guilin 541004, China; gxsd307@126.com

* Correspondence: xuweifeng_u@163.com (W.-F.X.); yang_rui_yun@163.com (R.-Y.Y.); zgh1207@gxnu.edu.cn (G.-H.Z.)

† These authors contributed equally to this work.

List of supporting information

Table S1. The NMR data (400 MHz for ^{1}H and 100 MHz for ^{13}C in DMSO-d_6) of **1** and **2**
Table S2. The NMR data (400 MHz for ^{1}H and 100 MHz for ^{13}C in $CDCl_3$) of **3** and **5**
Table S3. The NMR data (400 MHz for ^{1}H and 100 MHz for ^{13}C in DMSO-d_6) of **6** and **9**
Table S4. X-ray crystallographic data of compound **5**
Table S5. X-ray crystallographic data of compound **6**
Figure S1. The cell viability of BV-2 cells treated with **1–11**
Figure S2. The nitric oxide inhibition of BV-2 cells treated with **1–11**
Figure S3. ^{1}H NMR (400 MHz, Pyridine-d_5) spectrum of **1**
Figure S4. ^{13}C NMR (100 MHz, Pyridine-d_5) spectrum of **1**
Figure S5. HMQC (Pyridine-d_5) spectrum of **1**
Figure S6. ^{1}H-^{1}H COSY (Pyridine-d_5) spectrum of **1**
Figure S7. HMBC (Pyridine-d_5) spectrum of **1**
Figure S8. *J*-HMBC (Pyridine-d_5) spectrum of **1**
Figure S9. ^{1}H NMR (400 MHz, DMSO-d_6) spectrum of **1**
Figure S10. ^{13}C NMR (100 MHz, DMSO-d_6) spectrum of **1**
Figure S11. HMQC (DMSO-d_6) spectrum of **1**
Figure S12. ^{1}H-^{1}H COSY (DMSO-d_6) spectrum of **1**
Figure S13. HMBC (DMSO-d_6) spectrum of **1**
Figure S14. HRESIMS spectrum of **1**
Figure S15. ^{1}H NMR (400 MHz, DMSO-d_6) spectrum of **2**
Figure S16. ^{13}C NMR (100 MHz, DMSO-d_6) spectrum of **2**
Figure S17. HMQC (DMSO-d_6) spectrum of **2**
Figure S18. ^{1}H-^{1}H COSY (DMSO-d_6) spectrum of **2**
Figure S19. HMBC (DMSO-d_6) spectrum of **2**
Figure S20. HRESIMS spectrum of **2**
Figure S21. ^{1}H NMR (400 MHz, $CDCl_3$) spectrum of **3**
Figure S22. ^{13}C NMR (100 MHz, $CDCl_3$) spectrum of **3**
Figure S23. HMQC ($CDCl_3$) spectrum of **3**
Figure S24. ^{1}H-^{1}H COSY ($CDCl_3$) spectrum of **3**
Figure S25. HMBC ($CDCl_3$) spectrum of **3**
Figure S26. NOESY ($CDCl_3$) spectrum of **3**
Figure S27. HRESIMS spectrum of **3**
Figure S28. ^{1}H NMR (400 MHz, DMSO-d_6) spectrum of **6**
Figure S29. ^{13}C NMR (100 MHz, DMSO-d_6) spectrum of **6**
Figure S30. HMQC (DMSO-d_6) spectrum of **6**
Figure S31. ^{1}H-^{1}H COSY (DMSO-d_6) spectrum of **6**
Figure S32. HMBC (DMSO-d_6) spectrum of **6**
Figure S33. NOESY (DMSO-d_6) spectrum of **6**
Figure S34. HRESIMS spectrum of **6**
Figure S35. ^{1}H NMR (400 MHz, DMSO-d_6) spectrum of **7**
Figure S36. ^{13}C NMR (100 MHz, DMSO-d_6) spectrum of **7**
Figure S37. ^{1}H NMR (400 MHz, $CDCl_3$) spectrum of **7**

Figure S38. ^{13}C NMR (100 MHz, $CDCl_3$) spectrum of **7**
Figure S39. HMQC ($CDCl_3$) spectrum of **7**
Figure S40. ^{1}H-^{1}H COSY ($CDCl_3$) spectrum of **7**
Figure S41. HMBC ($CDCl_3$) spectrum of **7**
Figure S42. NOESY ($CDCl_3$) spectrum of **7**
Figure S43. HRESIMS spectrum of **7**
Figure S44. ^{1}H NMR (400 MHz, DMSO-d_6) spectrum of **8**
Figure S45. ^{13}C NMR (100 MHz, DMSO-d_6) spectrum of **8**
Figure S46. HMQC (DMSO-d_6) spectrum of **8**
Figure S47. ^{1}H-^{1}H COSY (DMSO-d_6) spectrum of **8**
Figure S48. HMBC (DMSO-d_6) spectrum of **8**
Figure S49. NOESY (DMSO-d_6) spectrum of **8**
Figure S50. HRESIMS spectrum of **8**
Figure S51. Map of location sites of sea sediment sample used in this study

Table S1. The NMR data (400 MHz for ^{1}H and 100 MHz for ^{13}C in DMSO-d_6) of **1** and **2**

No.	**1**		**2**	
	δ_C	δ_H (J in Hz)	δ_C	δ_H (J in Hz)
1	32.3	2.77, overlapped<br>2.48, overlapped	32.3	2.77, overlapped<br>2.48, overlapped
2	171.8		171.7	
4	37.4	2.77, overlapped	37.5	2.77, overlapped
5	50.5	2.90, d (2.8)	50.6	2.89, d (3.0)
6	76.9	4.72, t (2.8)	76.6	4.74, t (3.0)
7	40.4	2.64, overlapped<br>2.51, dd (15.2, 2.4)	39.7	2.64, overlapped<br>2.54, overlapped
8	142.5		142.4	
9	48.1	2.67, overlapped	48.0	2.67, overlapped
10	49.4		48.8	
11	25.0	1.70, m<br>1.40, m	24.8	1.70, m<br>1.40, m
12	38.8	2.25, m<br>1.96, m	38.8	2.25, m<br>1.96, m
13	158.5		158.4	
14	116.5	5.54, s	116.5	5.54, s
15	167.6		167.5	
16	18.4	2.05, s	18.3	2.05, s
17	115.4	5.03, s<br>4.88, s	115.3	5.03, s<br>4.88, s
18	176.2		174.9	
19	12.8	0.94, d (7.2)	13.0	0.96, d (7.2)
20	177.5		177.2	
21			52.2	3.62, s

Table S2. The NMR data (400 MHz for ^{1}H and 100 MHz for ^{13}C in $CDCl_3$) of **3** and **5**

**No.**	**3**		**5**	
	δ_C	δ_H (*J* in Hz)	δ_C	δ_H (*J* in Hz)
1	37.4	3.12, d (15.0)<br>2.66, d (15.0)	38.0	2.37, d (15.0)<br>2.54, d (15.0)
2	170.9		172.5	
4	37.8	2.86, q (7.4)	38.6	2.94, q (7.4)
5	51.7	2.46, dd (11.4, 7.4)	52.1	2.69, dd (11.4, 7.4)
6	77.3	4.93, td (11.4, 4.8)	77.3	4.23, td (11.4, 4.8)
7	42.1	2.23, t (11.8)<br>3.03, dd (11.8, 4.8)	43.1	2.24, t (11.8)<br>2.98, dd (11.8, 4.8)
8	141.0		141.6	
9	48.0	2.35, m	47.6	2.30, m
10	50.7		41.9	
11	23.5	1.75, m<br>1.42, m	22.9	1.77, m<br>1.62, m
12	39.8	2.35, m<br>2.07, m	40.0	2.30, m<br>2.12, m
13	162.1		163.2	
14	115.4	5.67, s	116.5	5.52, s
15	171.2		177.5	
16	19.4	2.18, s	19.9	2.18, s
17	114.0	5.18, s<br>4.83, s	115.0	5.25, s<br>4.85, s
18	178.5		179.4	
19	10.1	1.06, d (7.4)	12.5	1.26, d (7.4)
20	171.7		18.4	0.87, s
21	52.2	3.66, s		
22	52.3	3.71, s		

Table S3. The NMR data (400 MHz for ^{1}H and 100 MHz for ^{13}C in DMSO-d_6) of **6** and **9**

NO.	**6**		**9**	
	δ_C	δ_H, (*J* in Hz)	δ_C	δ_H, (*J* in Hz)
1	74.7	4.16, d (4.2)	75.0	4.16, d (4.2)
2	35.2	2.56, m<br>2.68, dd (15.8, 4.2)	29.0	2.42, m<br>2.70, dd (15.8, 4.2)
3	170.0		170.1	
4	79.6		80.0	
5	50.5	1.57, d (8.2)	50.5	1.67, m
6	25.2	1.52, m	22.5	1.69, m
7	69.2	3.69, m	72.9	4.76, dt, (10.8, 5.2)
8	49.1	2.19, m	49.3	2.53, m
9	86.9		86.6	
10	49.5		49.9	
11	48.8	1.86, d (14.2)<br>2.41, d (14.2)	49.1	1.88, d (14.2)<br>2.55, d (14.2)
12	10.3	0.87, d (7.4)	10.8	0.91, d (7.4)
13	65.5	4.45, d (13.0)<br>4.52, d (13.0)	65.6	4.45, d (13.0)<br>4.65, d (13.0)
14	21.9	0.98, s	21.3	1.00, s
15	30.9	1.18, s	30.7	1.20, s
16			170.2	
17			21.2	2.01, s
1′	46.8		47.0	
2′	127.8		128.1	
3′	107.3		107.7	
4′	78.2	5.04, d (8.4)	78.6	5.04, d (8.4)
5′	41.3	2.83, dd (8.4, 6.2)	41.6	2.84, dd (8.4, 6.2)
6′	38.9	2.07, m	39.3	2.05, m
7′	18.4	0.74, s	18.6	0.75, s
8′	21.9	1.62, s	22.2	1.62, s
9′	175.1		175.3	
10′	11.8	1.04, d (7.4)	12.2	1.05, d (7.4)

Table S4. X-ray crystallographic data of compound **5**

Identification code	**5**
Empirical formula	$C_{19}H_{23}O_6$
Formula weight	347.37
Temperature [K]	297.0 (2)
Crystal system	monoclinic
Space group (number)	*C*2y
a [Å]	28.1075(12)
b [Å]	6.8034(2)
c [Å]	9.8758(4)
α [°]	90
β [°]	96.987(4)
γ [°]	90
Volume [Å^3]	1874.49(12)
Z	4
ρ_{calc} [gcm^{-3}]	1.231
μ [mm^{-1}]	0.757
F(000)	742.7
Radiation	CuK_α (λ=1.54184)
2θ range [°]	6.34 to 154.17
Index ranges	$-34 \leq h \leq 34$, $-5 \leq k \leq 8$, $-12 \leq l \leq 12$
Reflections collected	6689
Independent reflections	2694 [R_{int} = 0.0292, R_{sigma} = 0.0257]
Completeness to θ = 67.684°	99.9 %
Data / Restraints / Parameters	2694/1/239
Goodness-of-fit on F^2	1.049
Final *R* indexes [$I \geq 2\sigma(I)$]	R_1 = 0.0418, wR_2 = 0.1231
Final *R* indexes [all data]	R_1 = 0.0484, wR_2 = 0.1297
Flack parameter	0.12(17)

Table S5. X-ray crystallographic data of compound **6**

Identification code	**6**
Empirical formula	$C_{25}H_{34}O_9$
Formula weight	478.22
Temperature [K]	297.2(6)
Crystal system	orthorhombic
Space group (number)	$\boldsymbol{P2_12_12_1}$
a [Å]	10.86785(12)
b [Å]	14.38306(19)
c [Å]	30.5354(4)
α [°]	90.0
β [°]	90.0
γ [°]	90.0
Volume [Å^3]	4773.07(11)
Z	4
ρ_{calc} [gcm^{-3}]	1.332
μ [mm^{-1}]	0.84
F(000)	2048.0
Radiation	Cu K_α (λ=1.54184)
2θ range [°]	6.79 to 154.99
Index ranges	$-6 \leq h \leq 13$, $-18 \leq k \leq 16$, $-38 \leq l \leq 23$
Reflections collected	19923
Independent reflections	5177 [R_{int} = 0.0357, R_{sigma} = 0.0413]
Completeness to θ = 66.968°	99.5 %
Data / Restraints / Parameters	8666/1/628
Goodness-of-fit on F^2	10.6387
Final *R* indexes [$I \geq 2\sigma(I)$]	R_1 = 0.0362, wR_2 = 0.0911
Final *R* indexes [all data]	R_1 = 0.0439, wR_2 = 0.1102
Flack X parameter	0.06(9)

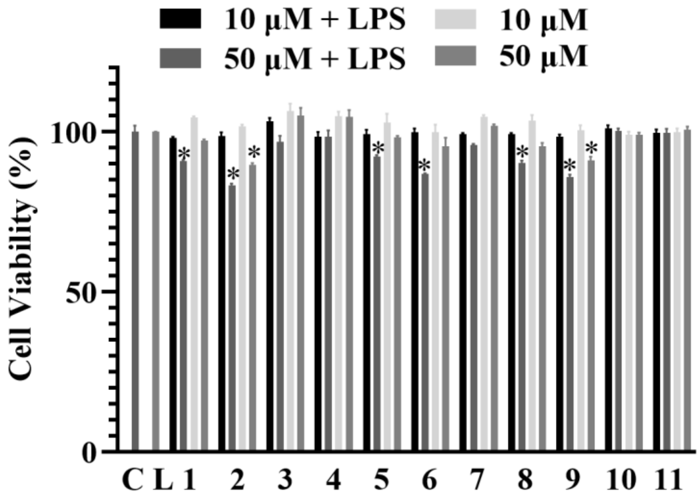

Figure S1. The cell viability of BV-2 cells treated with **1–11**

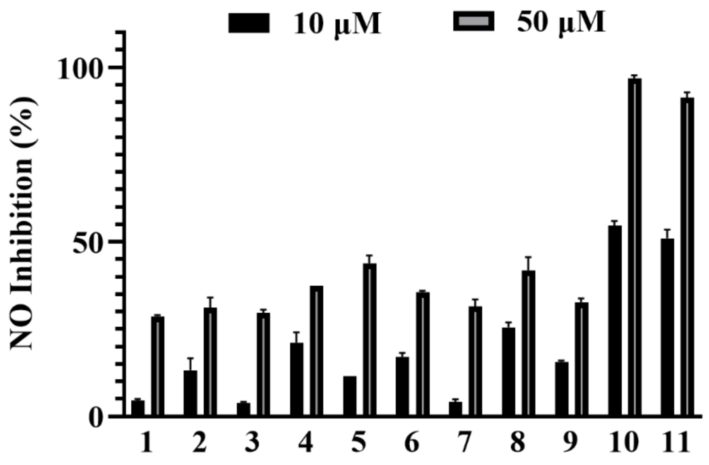

Figure S2. The nitric oxide inhibition of BV-2 cells treated with **1–11**

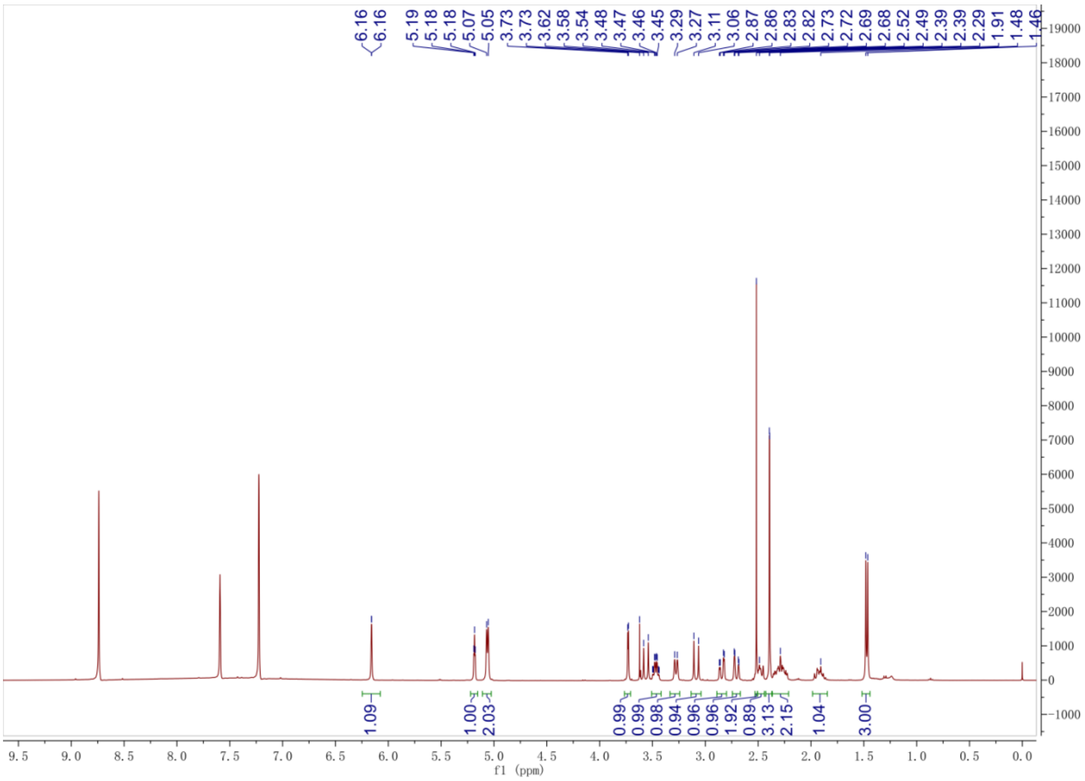

Figure S3. ^{1}H NMR (400 MHz, Pyridine-d_5) spectrum of **1**

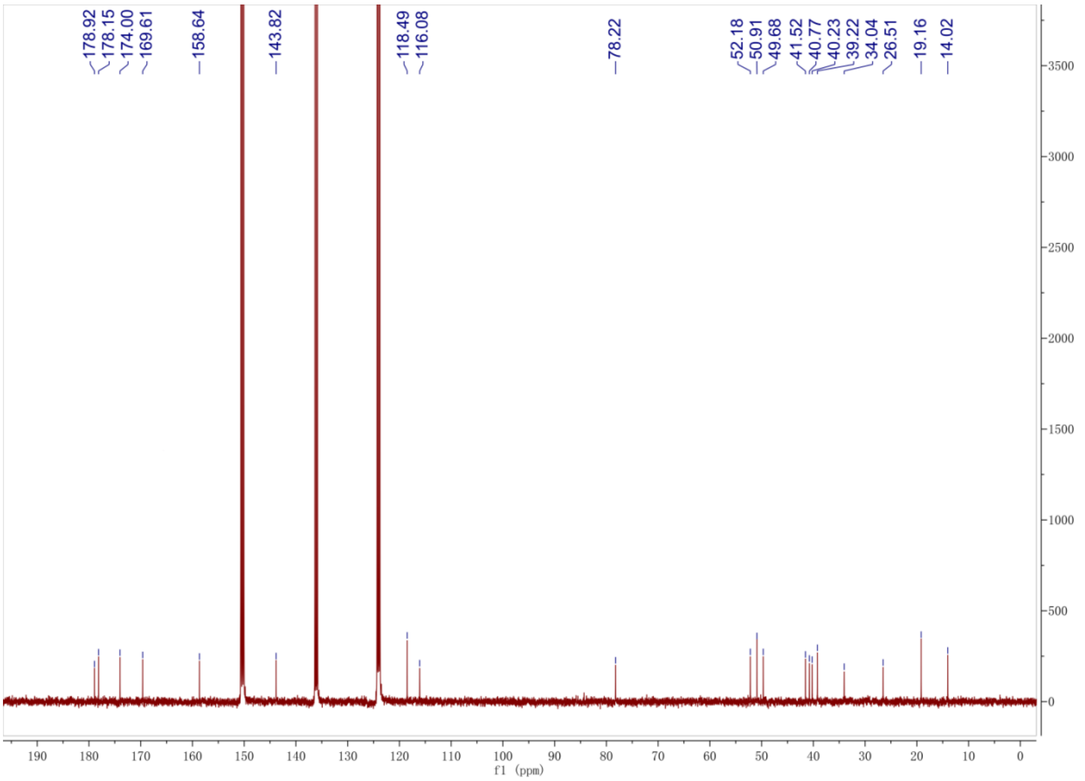

Figure S4. ^{13}C NMR (100 MHz, Pyridine-d_5) spectrum of **1**

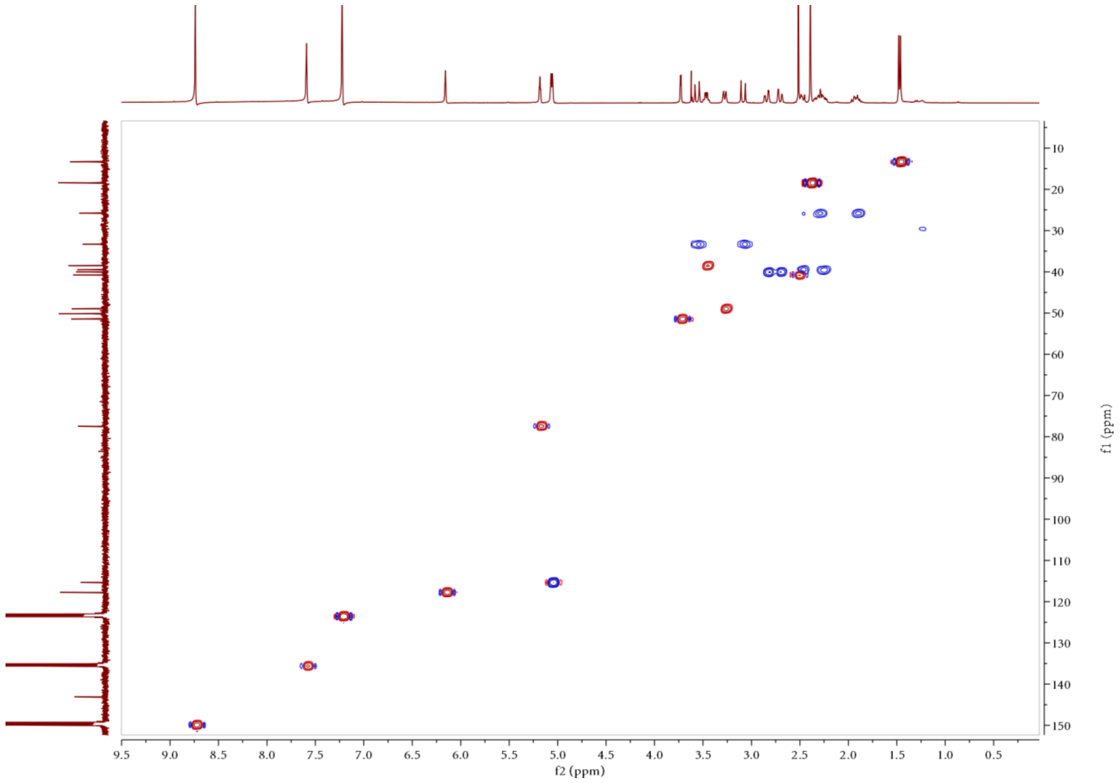

Figure S5. HMQC (Pyridine-d_5) spectrum of **1**

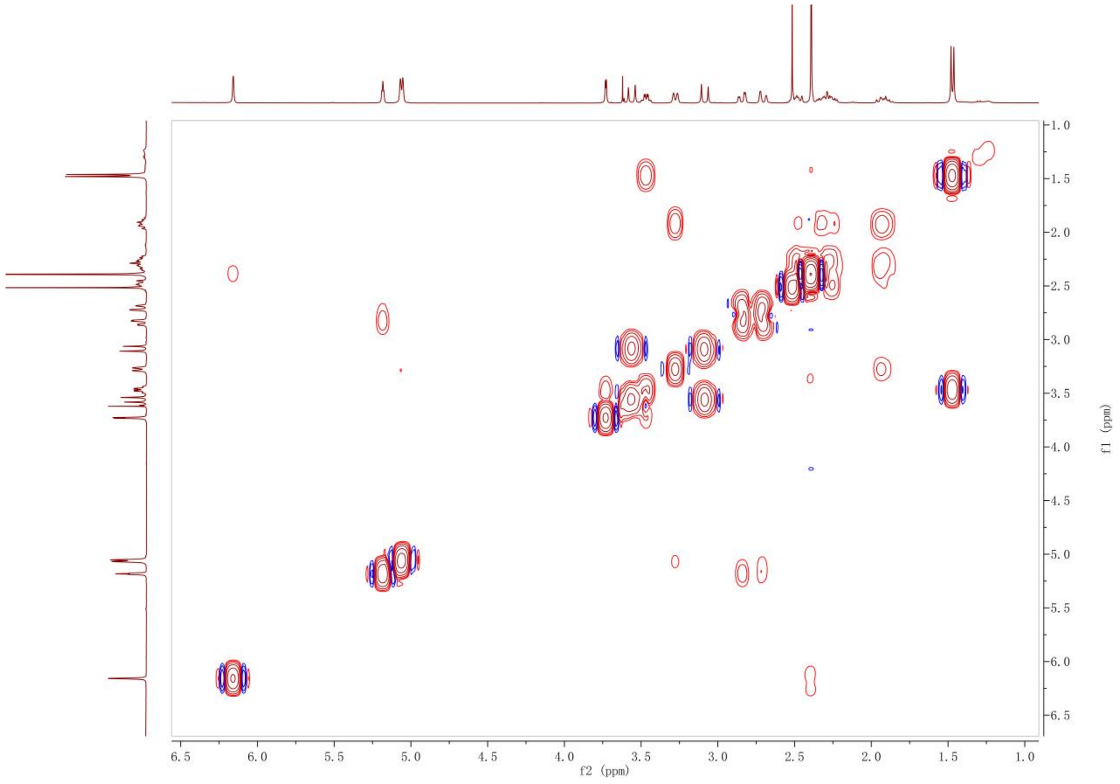

Figure S6. ^{1}H-^{1}H COSY (Pyridine-d_5) spectrum of **1**

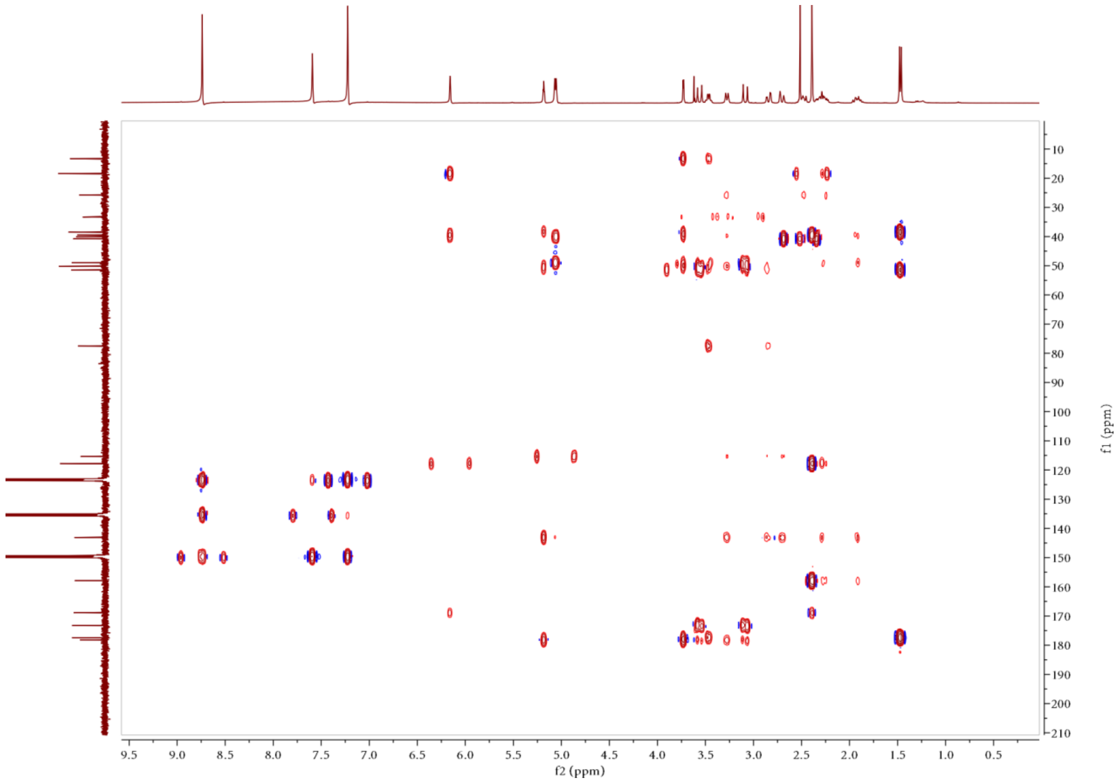

Figure S7. HMBC (Pyridine-d_5) spectrum of **1**

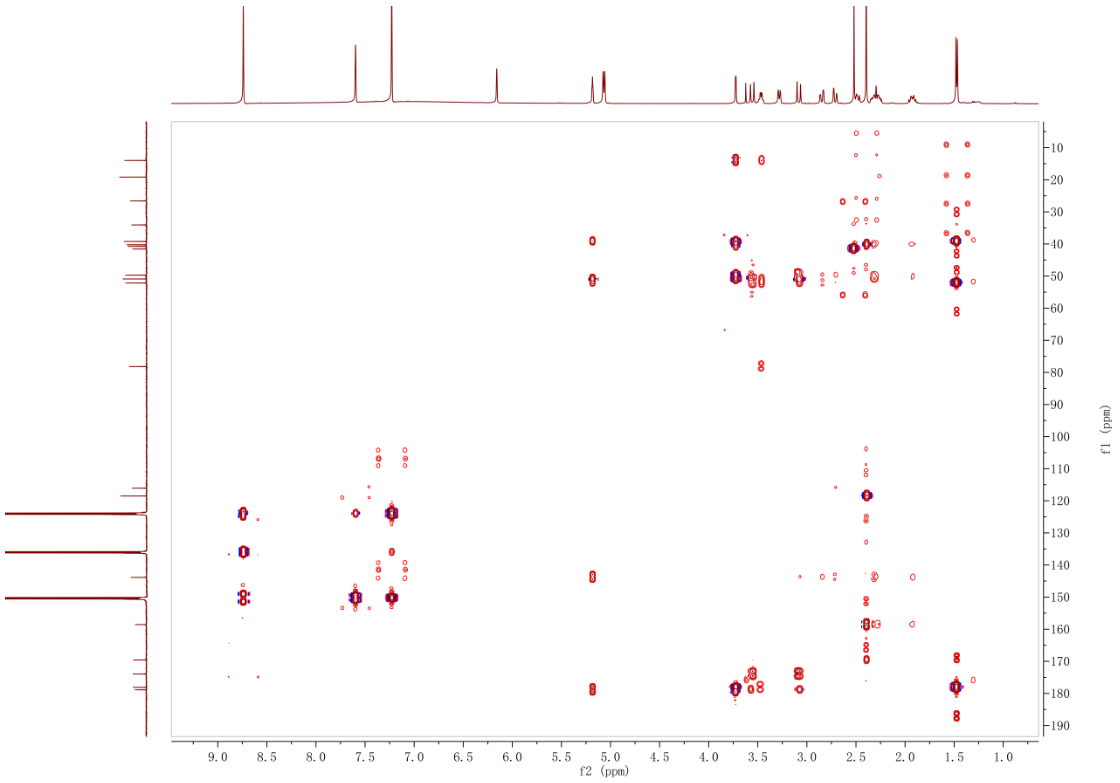

Figure S8. *J*-HMBC (Pyridine-d_5) spectrum of **1**

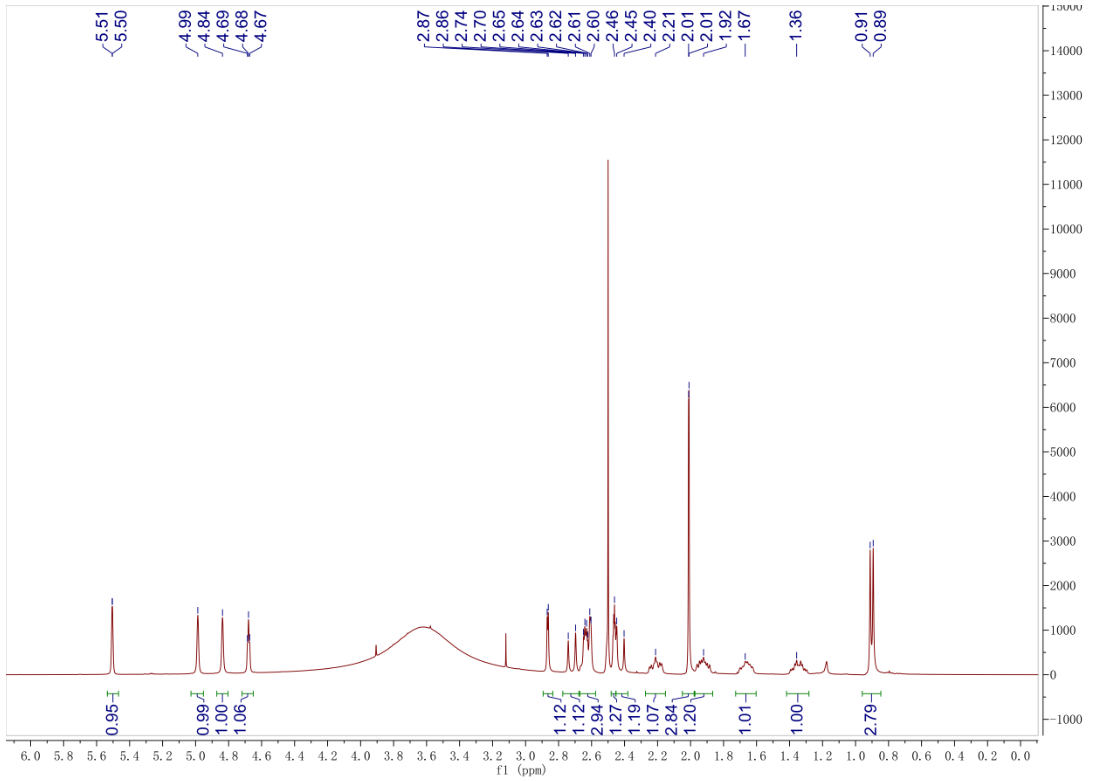

Figure S9. ^{1}H NMR (400 MHz, DMSO-d_6) spectrum of **1**

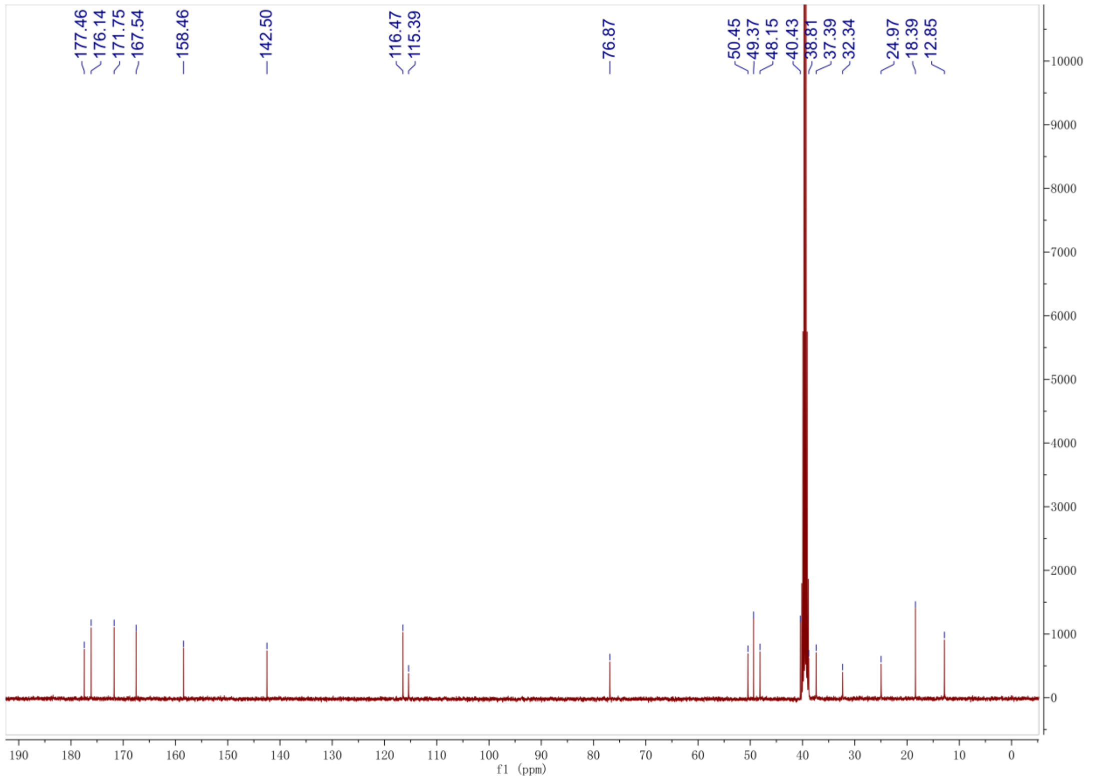

Figure S10. ^{13}C NMR (100 MHz, DMSO-d_6) spectrum of **1**

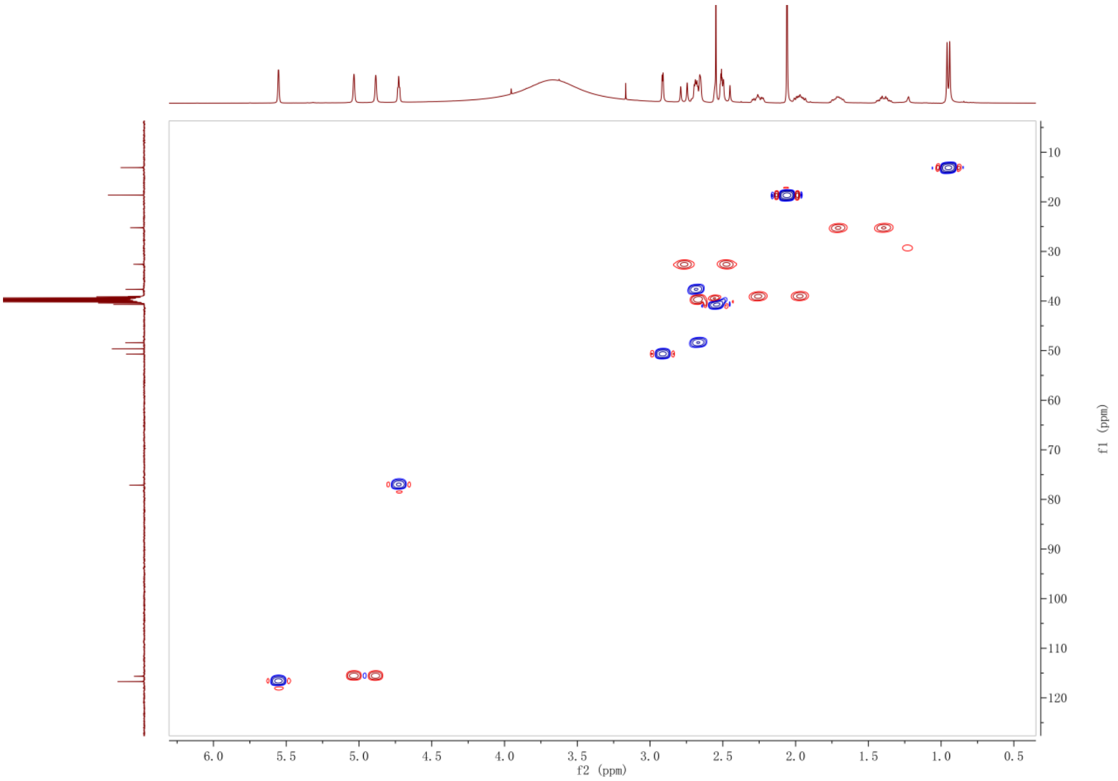

Figure S11. HMQC (DMSO-*d*6) spectrum of **1**

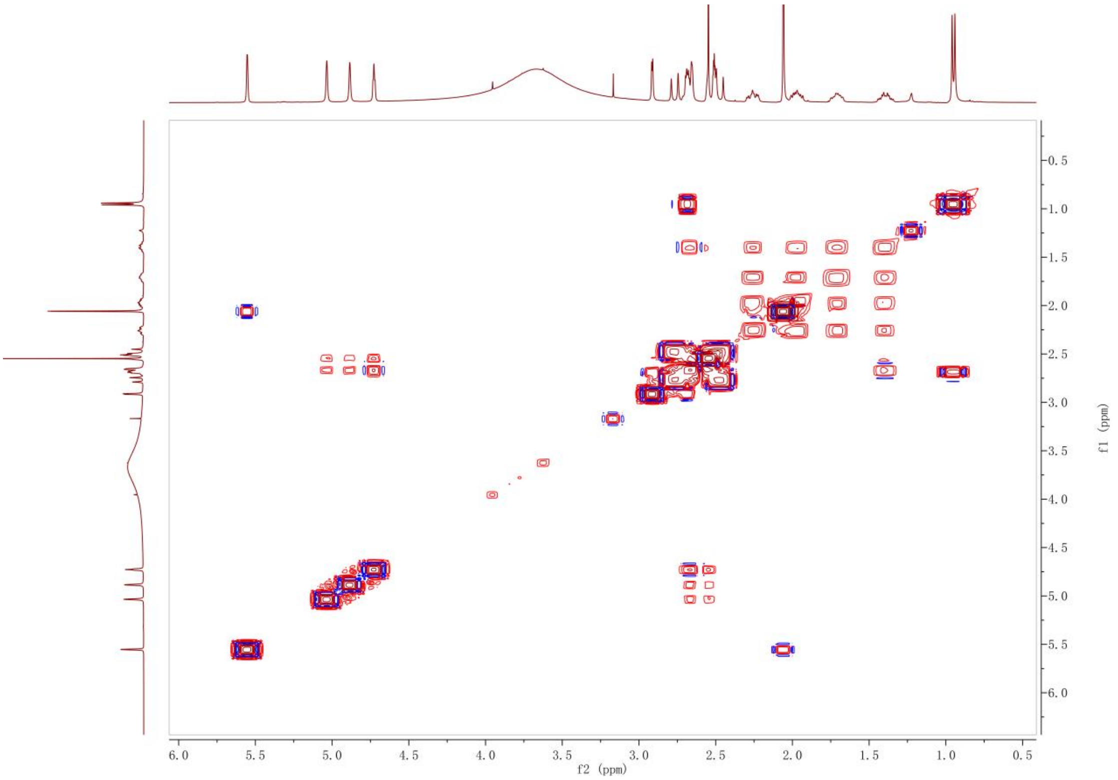

Figure S12. ^{1}H-^{1}H COSY (DMSO-*d*6) spectrum of **1**

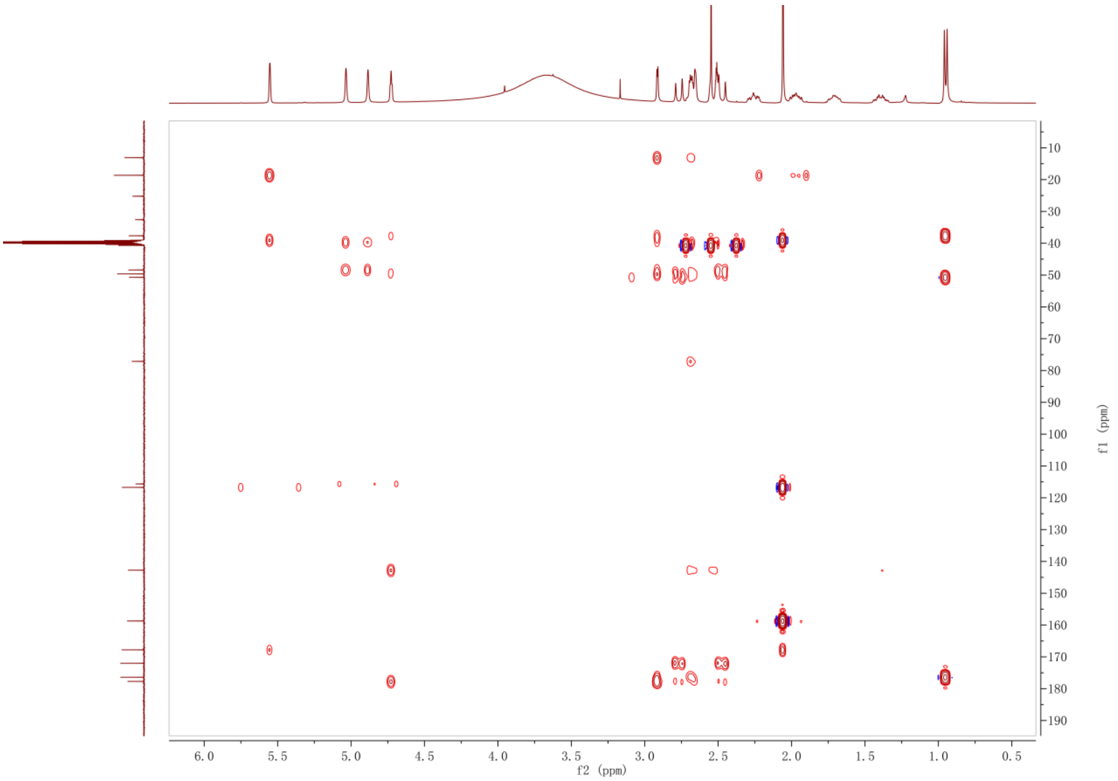

Figure S13. HMBC (DMSO-d_6) spectrum of **1**

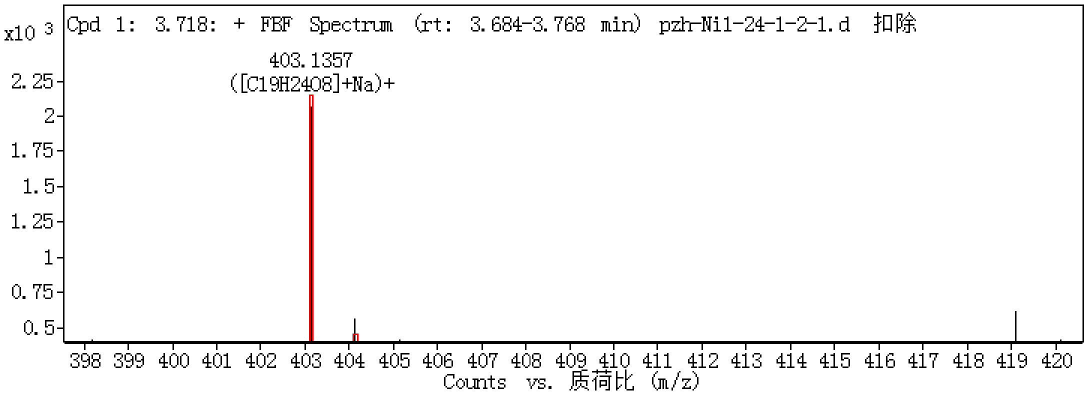

Figure S14. HRESIMS spectrum of **1**

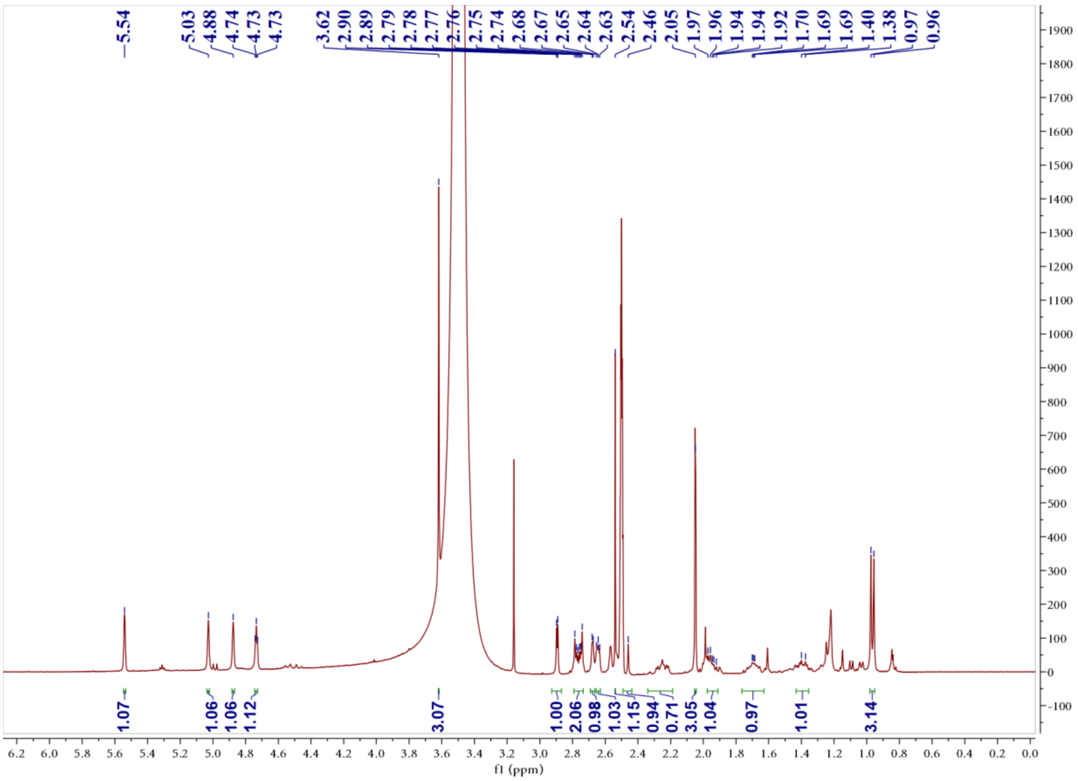

Figure S15. ^{1}H NMR (400 MHz, DMSO-*d*$_6$) spectrum of **2**

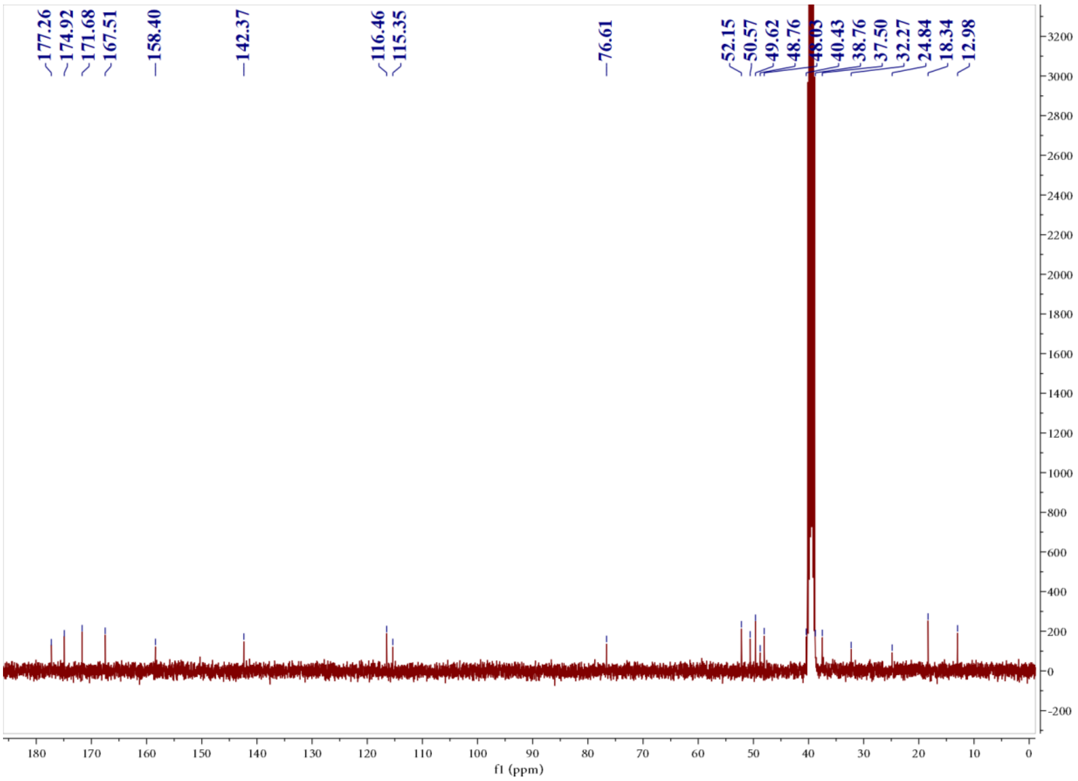

Figure S16. ^{13}C NMR (100 MHz, DMSO-*d*$_6$) spectrum of **2**

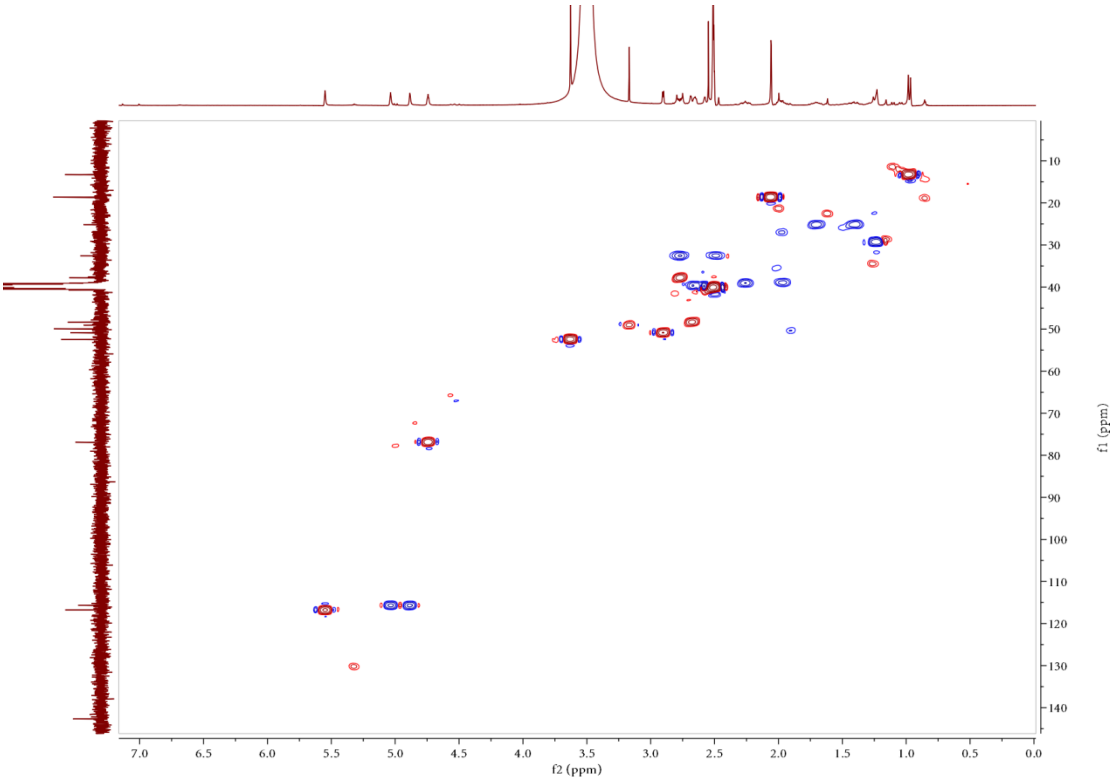

Figure S17. HMQC (DMSO-d_6) spectrum of **2**

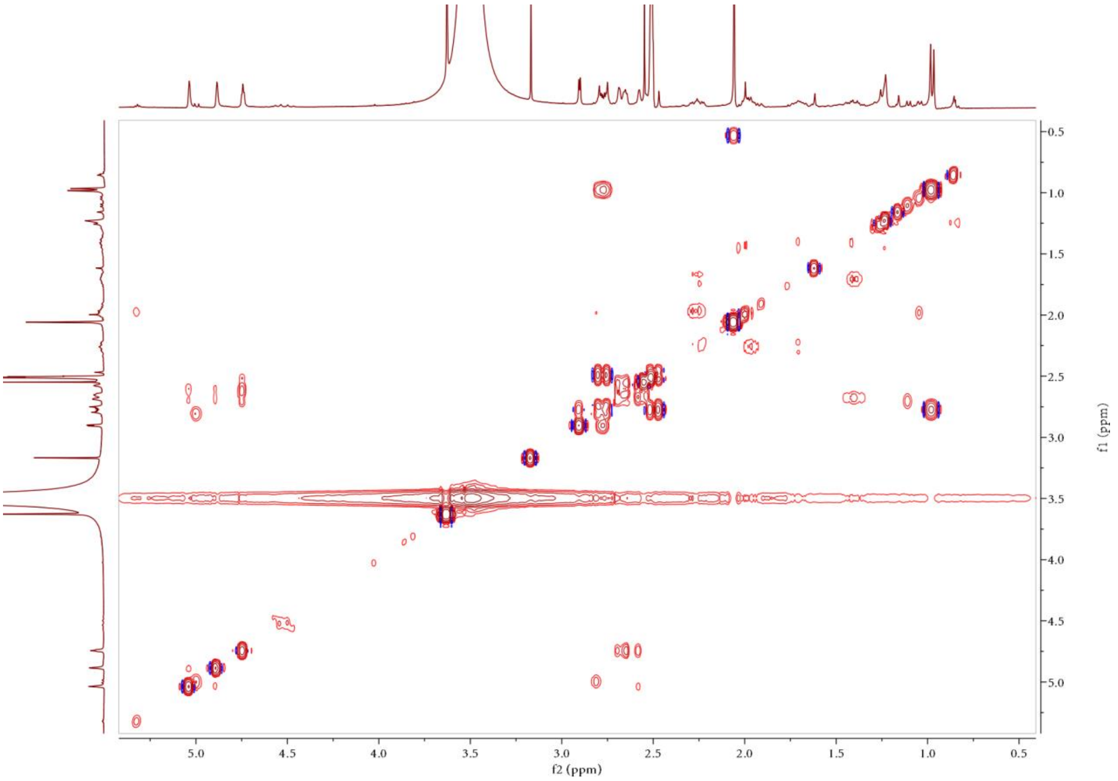

Figure S18. ^{1}H-^{1}H COSY (DMSO-d_6) spectrum of **2**

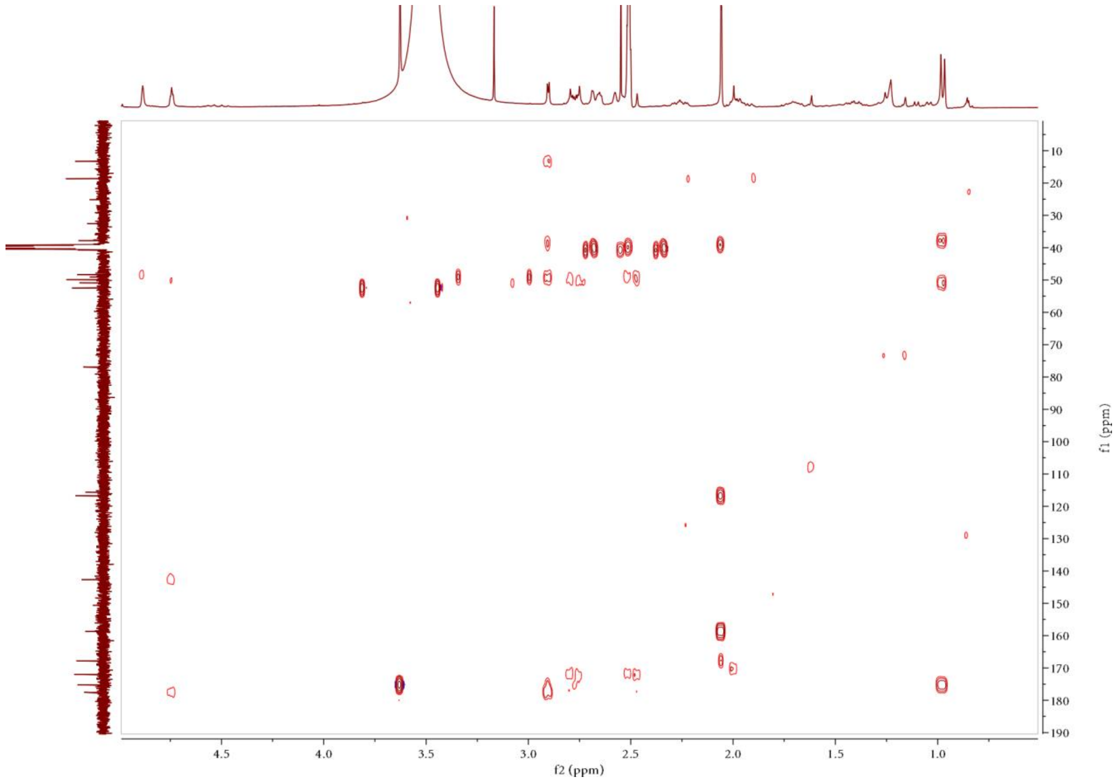

Figure S19. HMBC (DMSO-d_6) spectrum of **2**

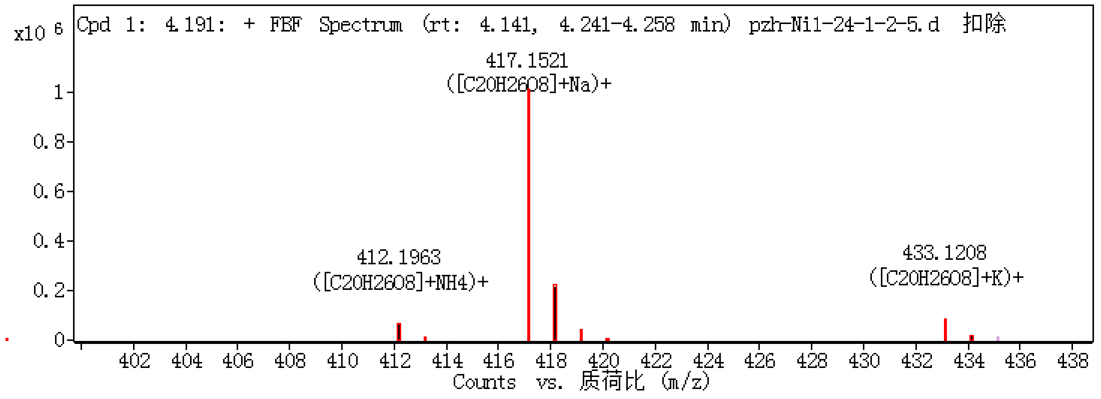

Figure S20. HRESIMS spectrum of **2**

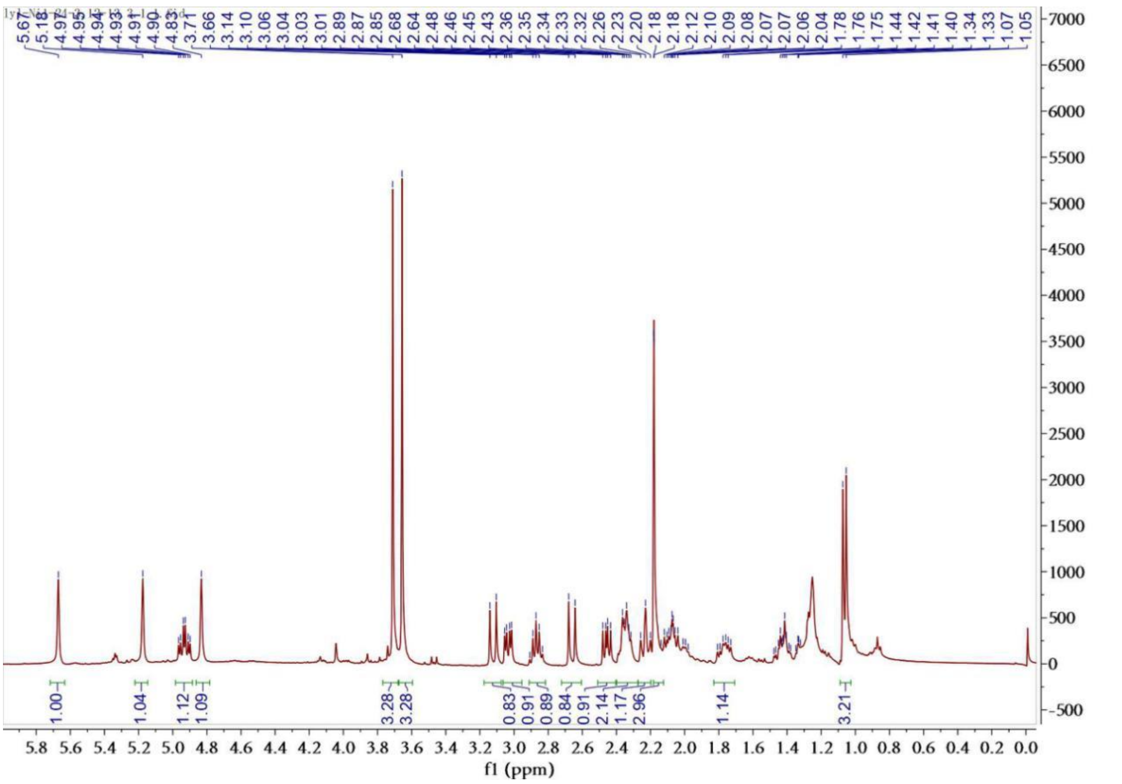

Figure S21. ^{1}H NMR (400 MHz, $CDCl_3$) spectrum of **3**

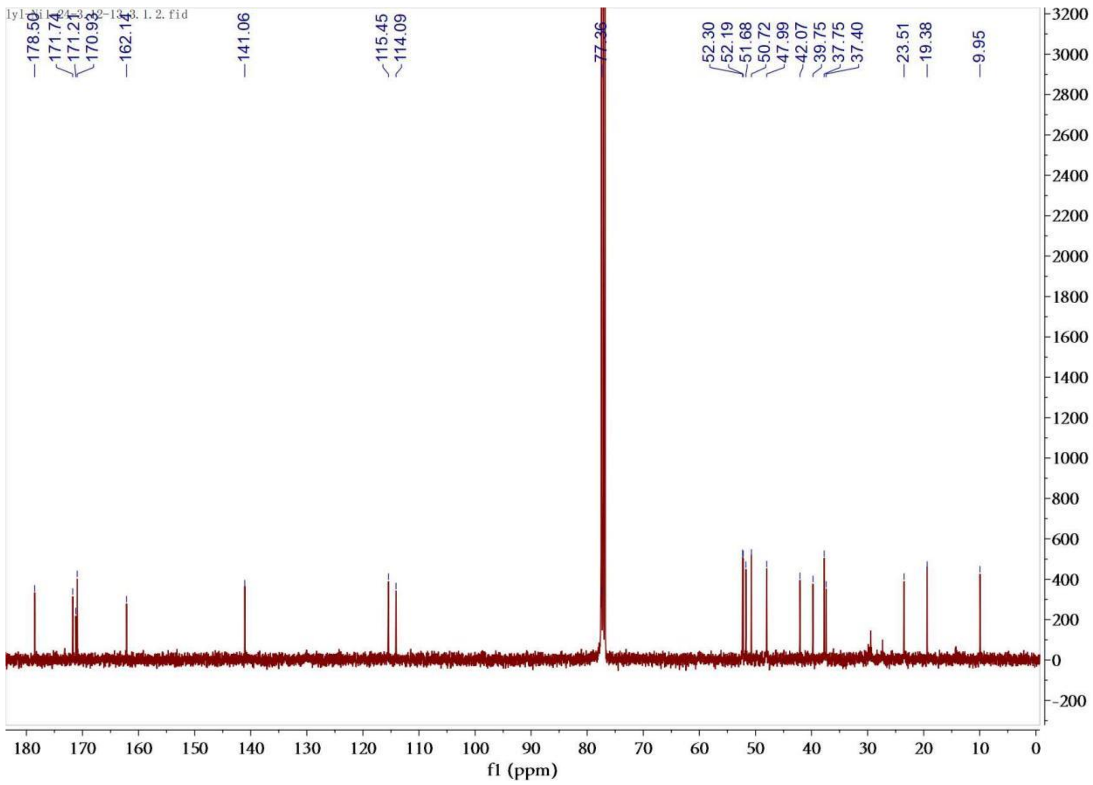

Figure S22. ^{13}C NMR (100 MHz, $CDCl_3$) spectrum of **3**

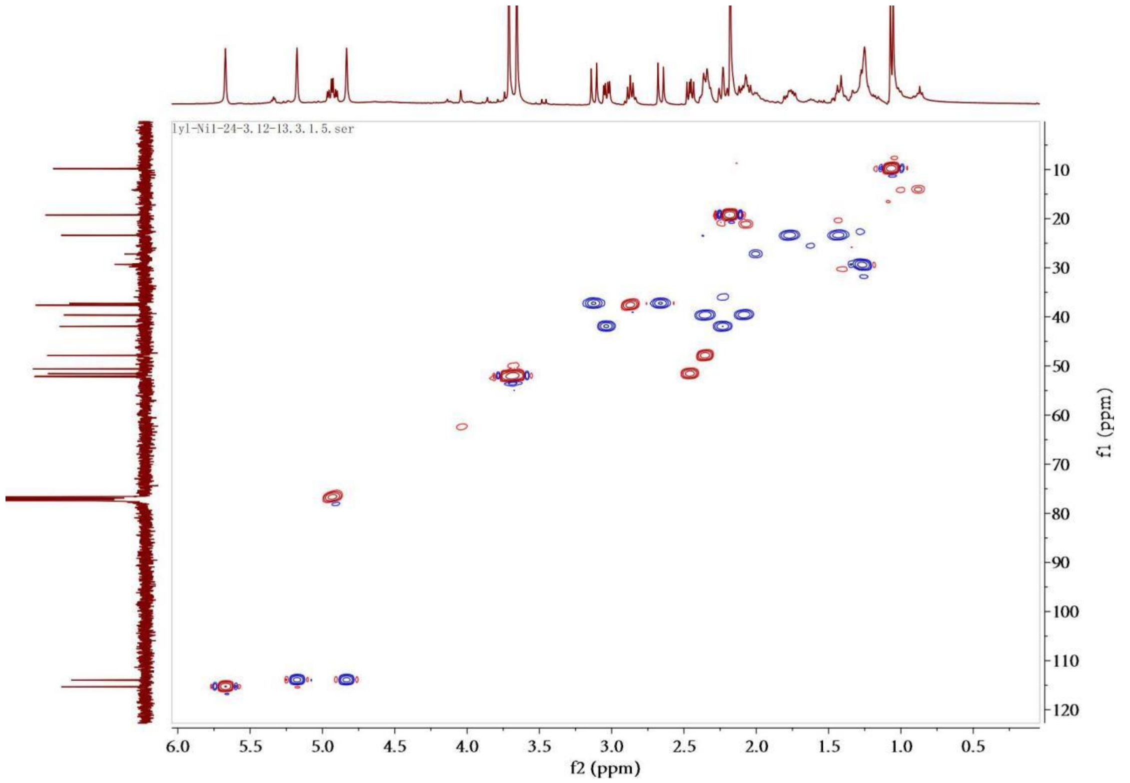

Figure S23. HMQC ($CDCl_3$) spectrum of **3**

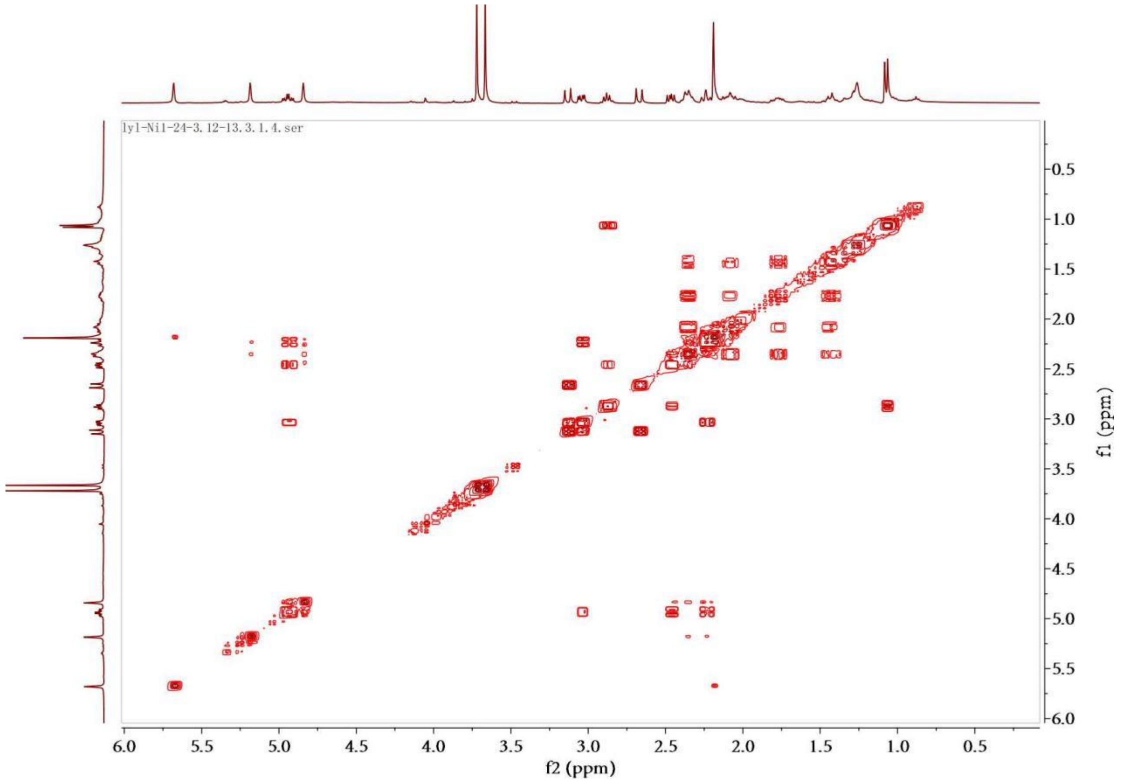

Figure S24. ^1H-^1H COSY ($CDCl_3$) spectrum of **3**

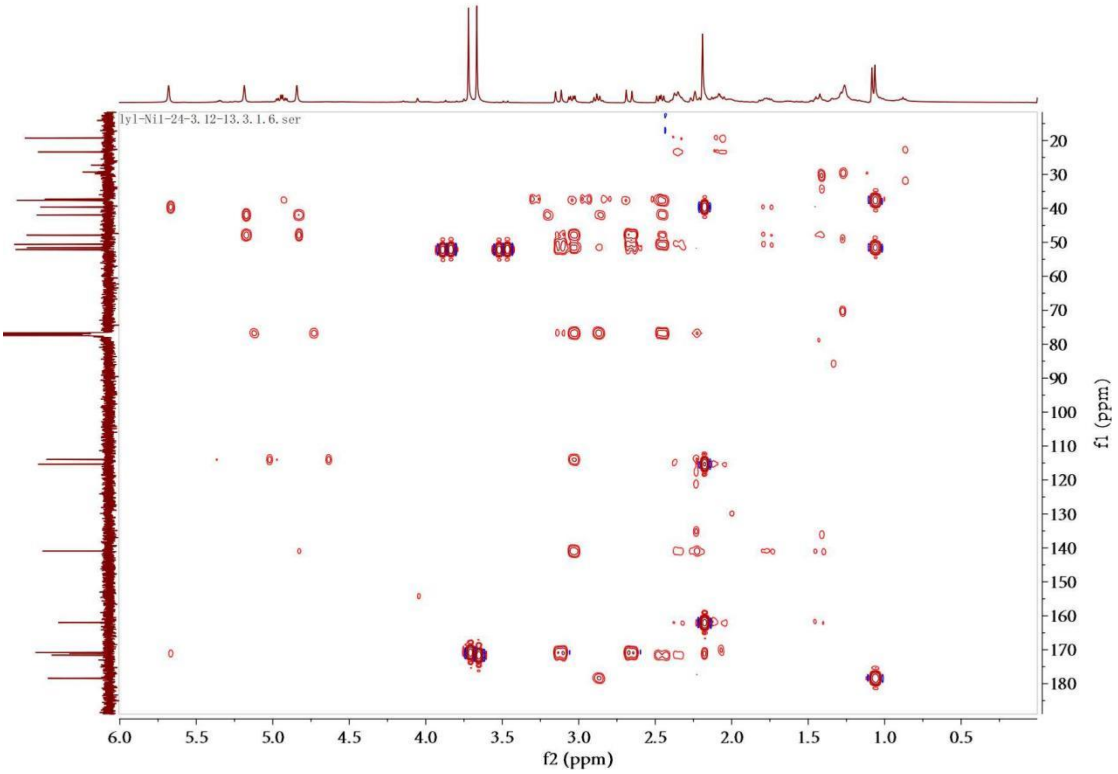

Figure S25. HMBC ($CDCl_3$) spectrum of **3**

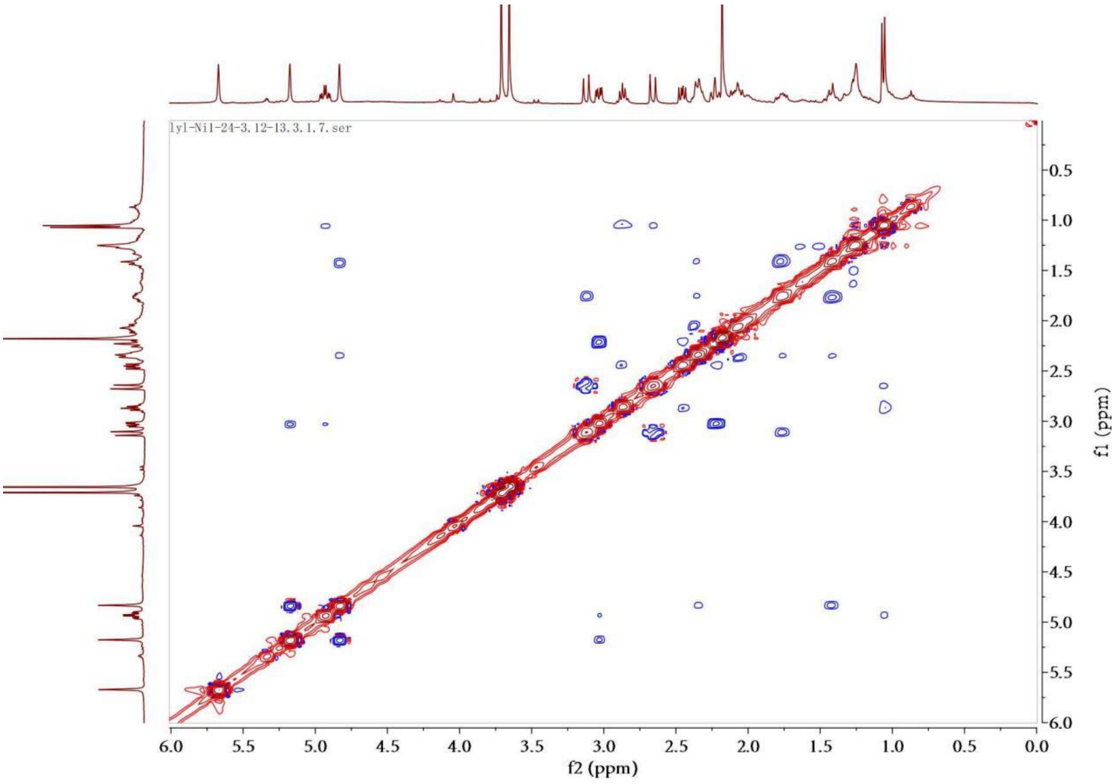

Figure S26. NOESY ($CDCl_3$) spectrum of **3**

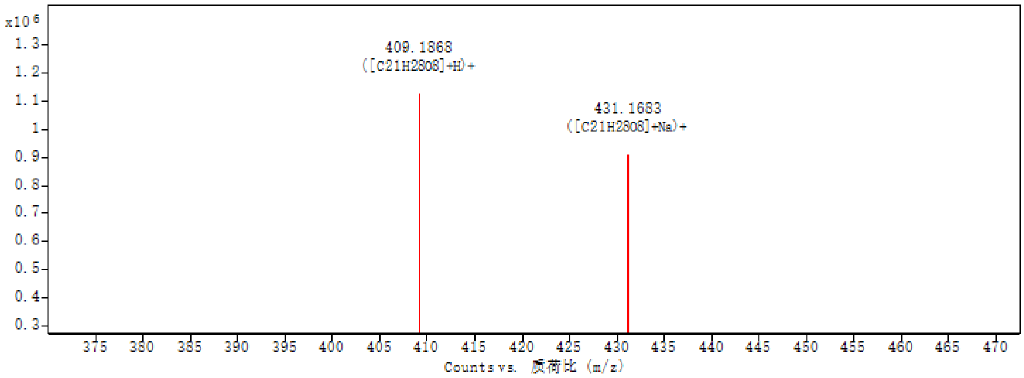

Figure S27. HRESIMS spectrum of **3**

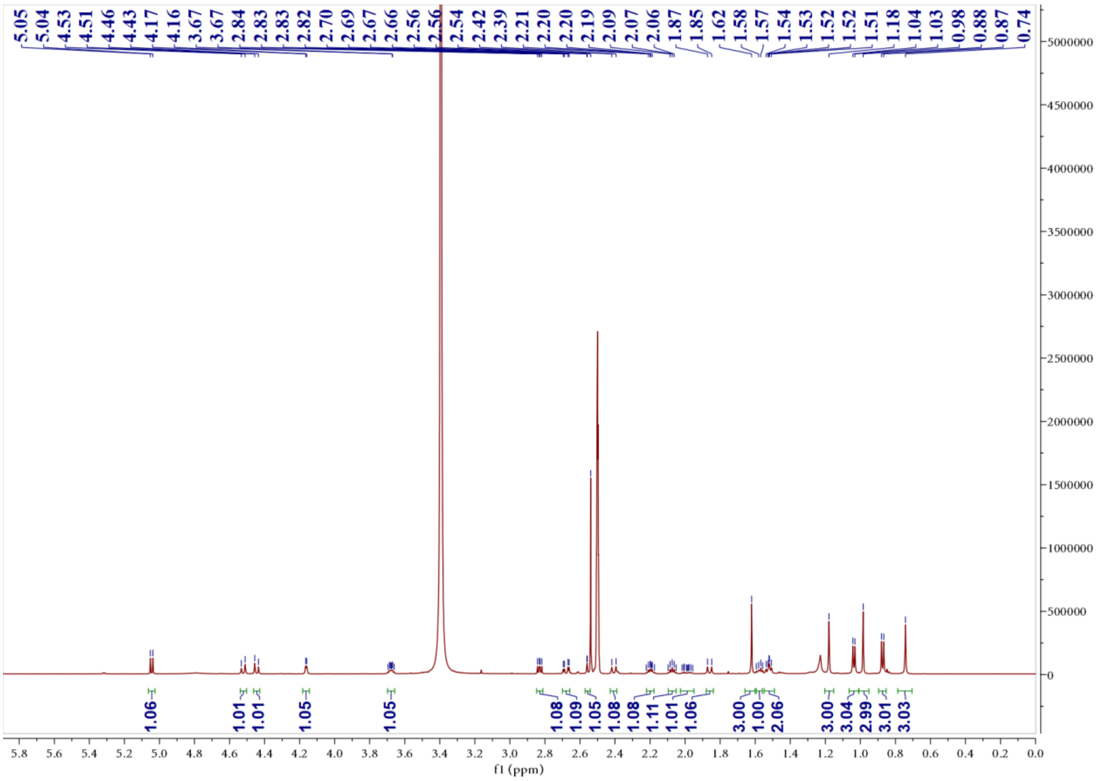

Figure S28. ^{1}H NMR (400 MHz, DMSO-d_6) spectrum of **6**

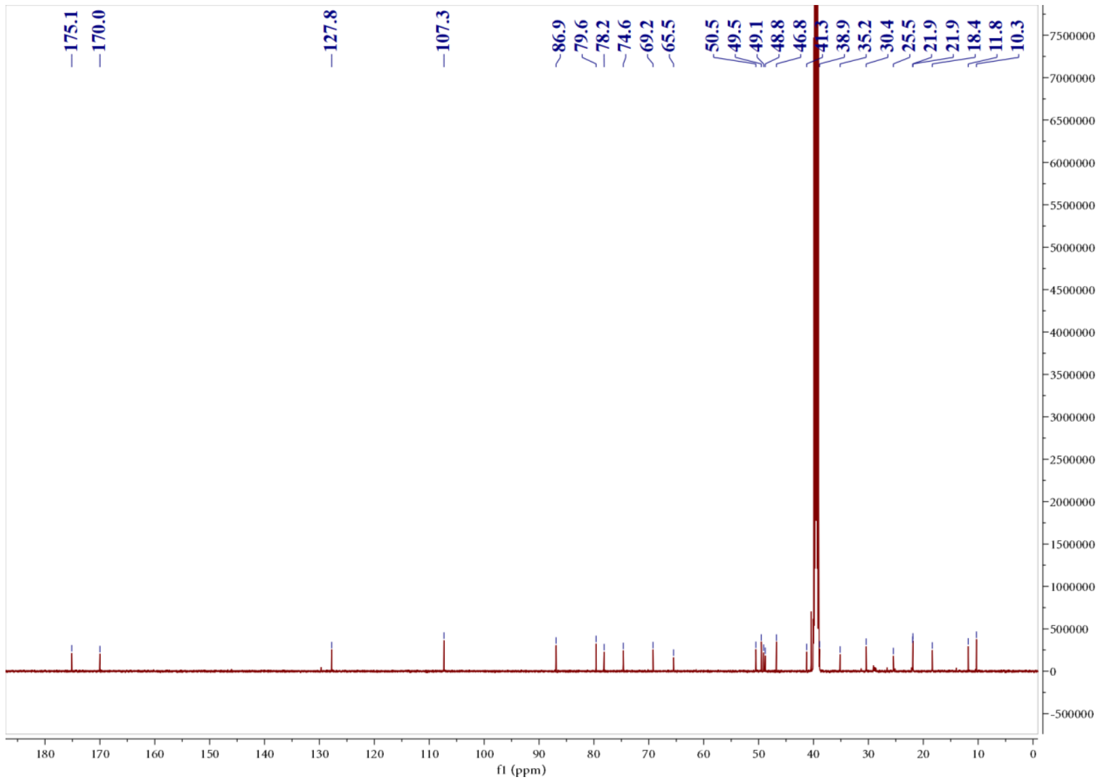

Figure S29. ^{13}C NMR (100 MHz, DMSO-d_6) spectrum of **6**

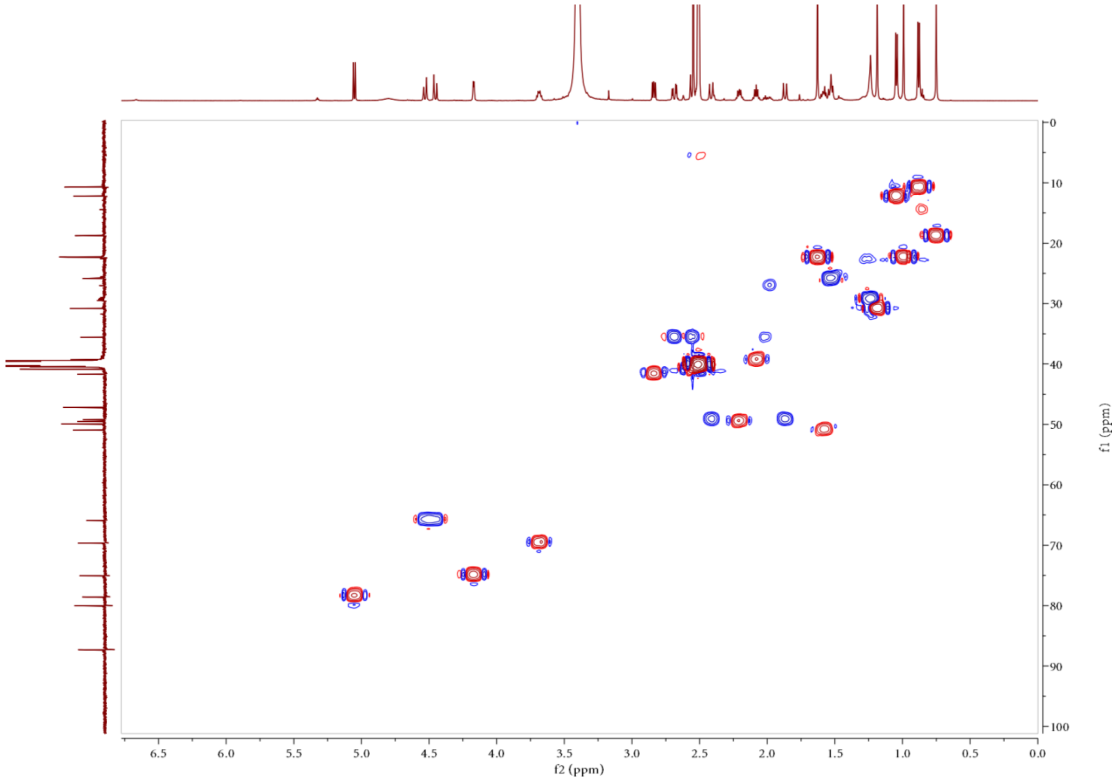

Figure S30. HMQC (DMSO-d_6) spectrum of **6**

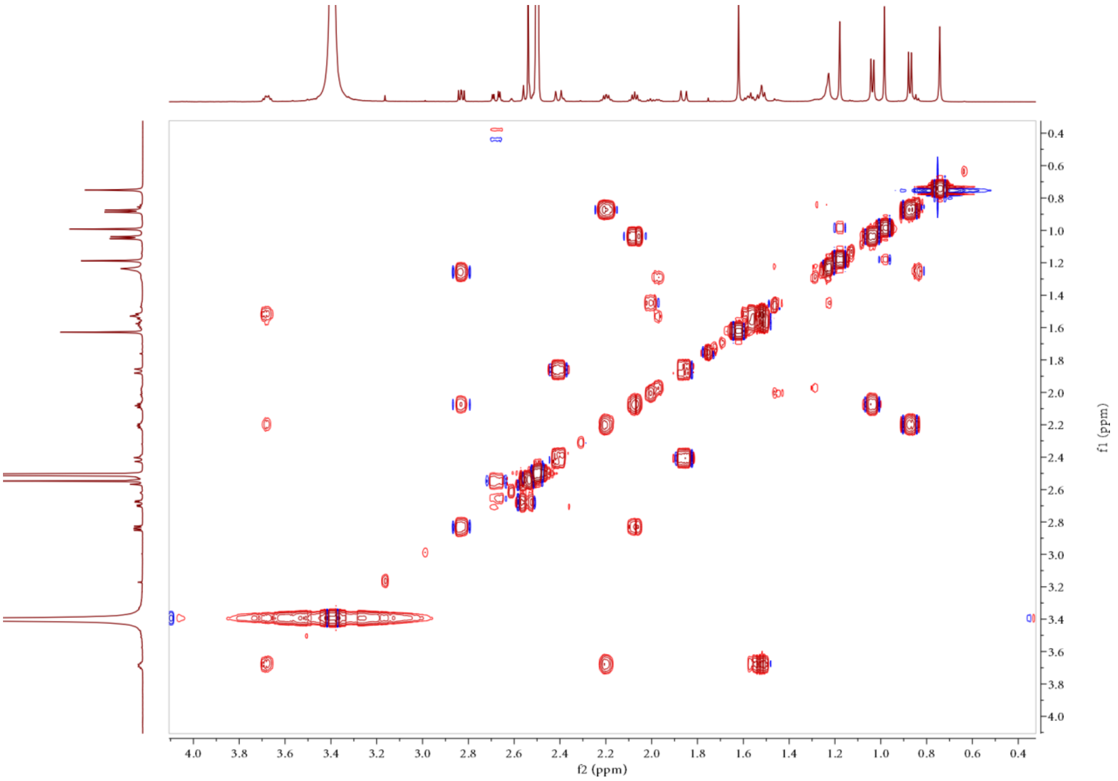

Figure S31. ^{1}H-^{1}H COSY (DMSO-d_6) spectrum of **6**

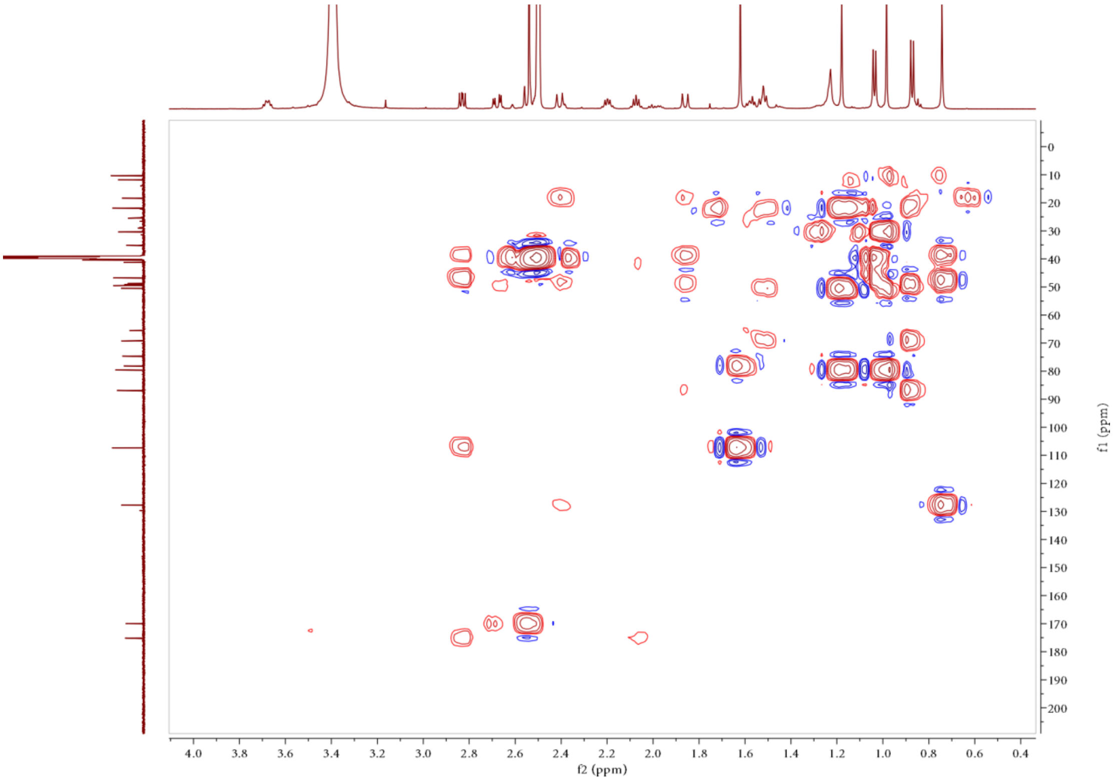

Figure S32. HMBC (DMSO-d_6) spectrum of **6**

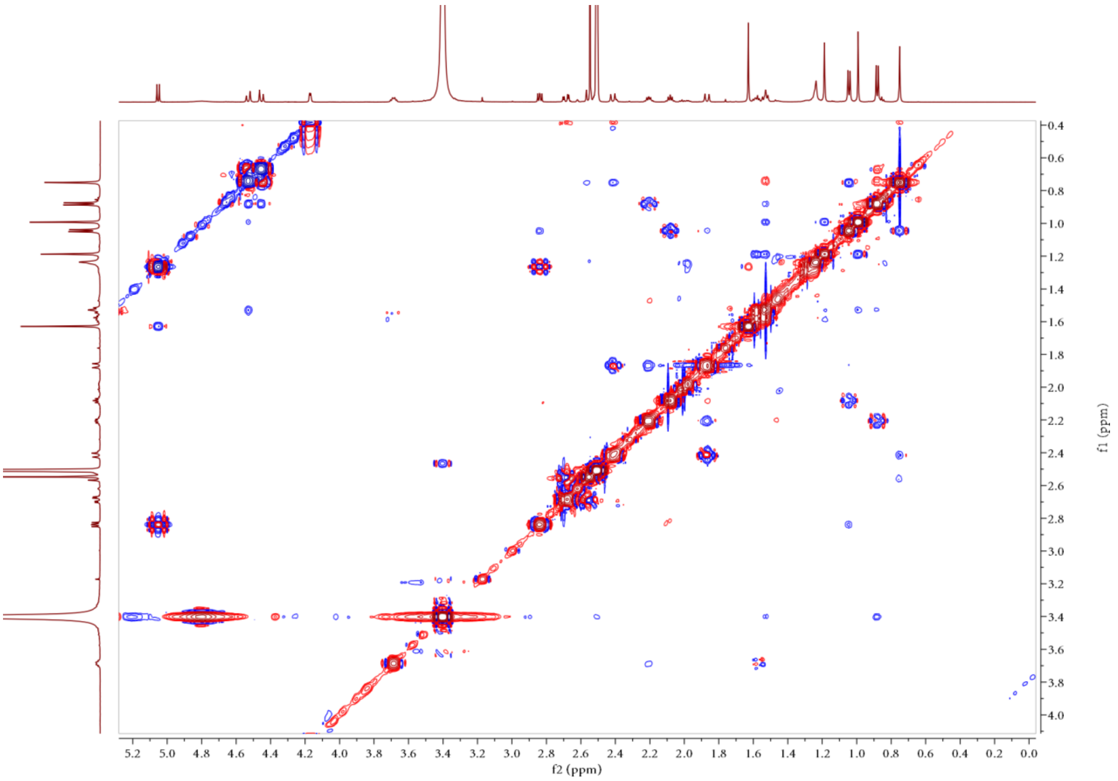

Figure S33. NOESY (DMSO-d_6) spectrum of **6**

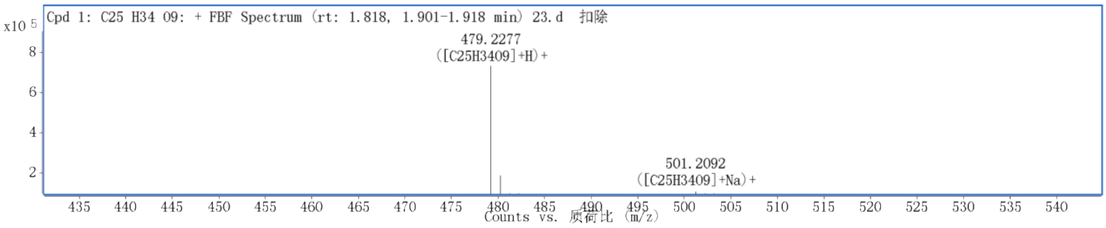

Figure S34. HRESIMS spectrum of **6**

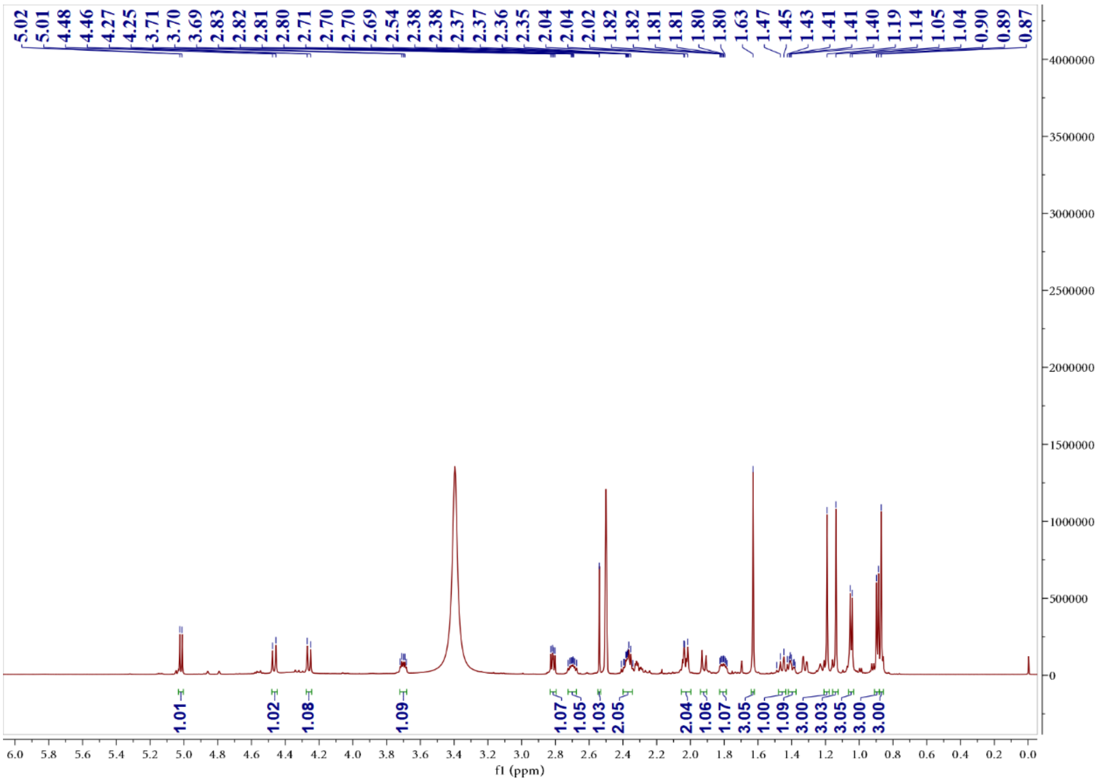

Figure S35. ^{1}H NMR (400 MHz, DMSO-*d*$_6$) spectrum of **7**

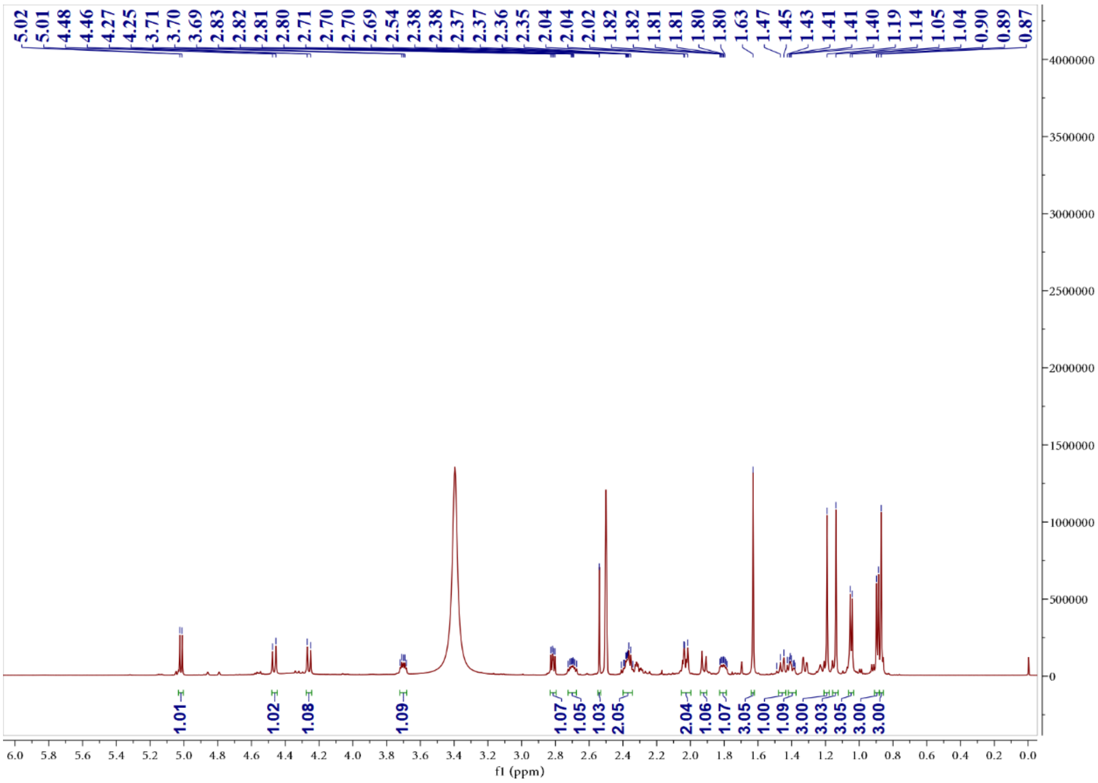

Figure S36. ^{13}C NMR (100 MHz, DMSO-*d*$_6$) spectrum of **7**

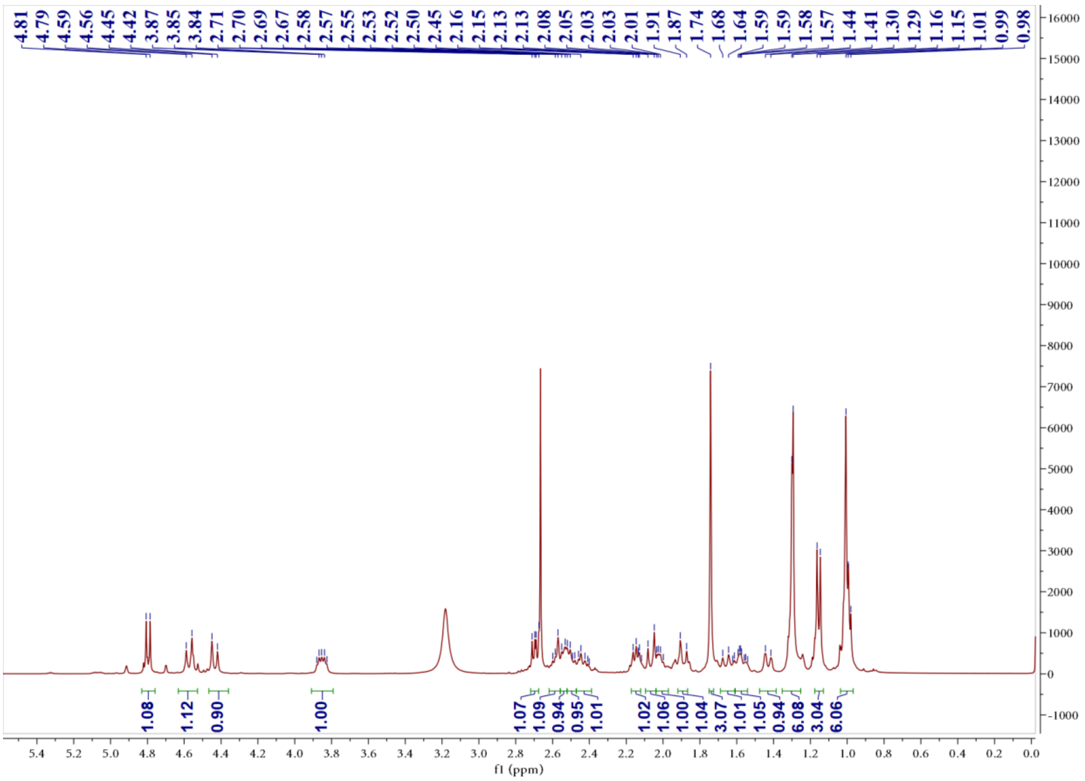

Figure S37. ^1H NMR (400 MHz, $CDCl_3$) spectrum of **7**

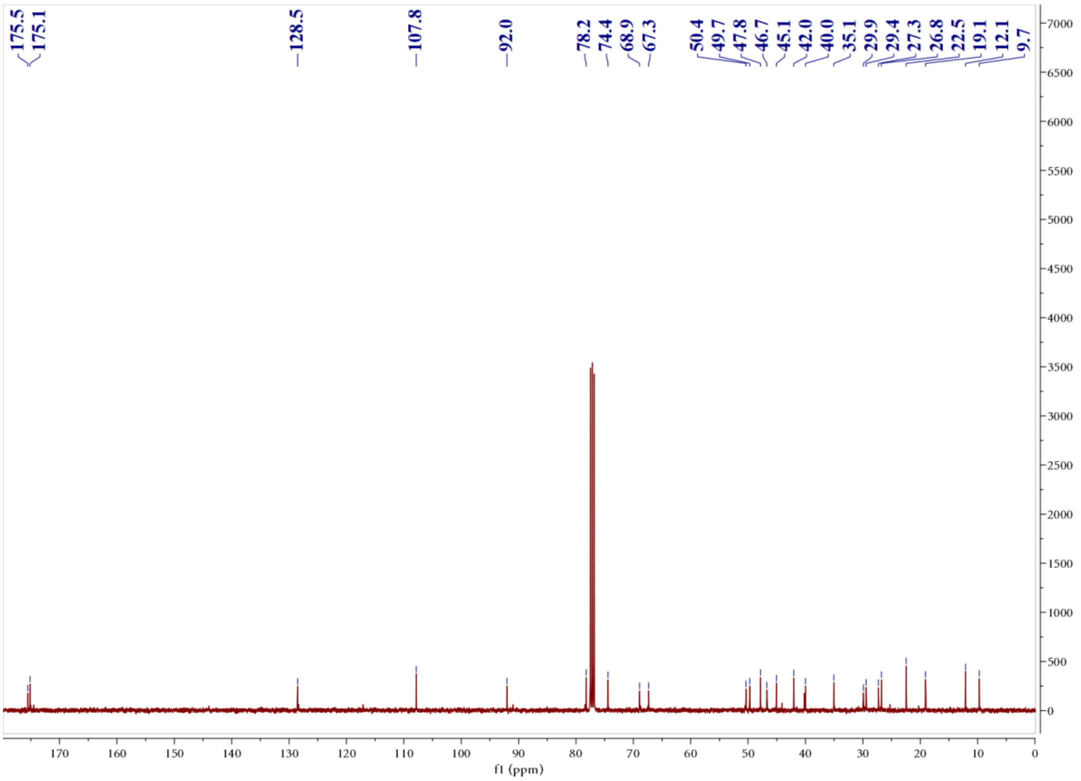

Figure S38. ^{13}C NMR (100 MHz, $CDCl_3$) spectrum of **7**

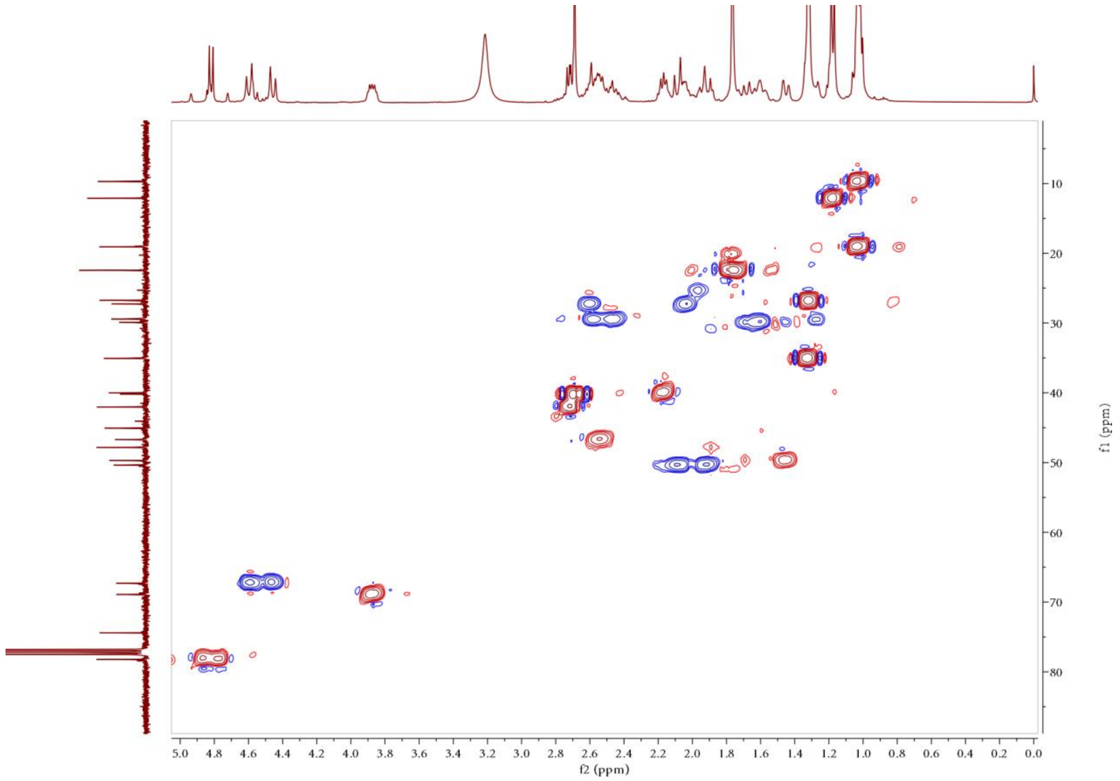

Figure S39. HMQC ($CDCl_3$) spectrum of **7**

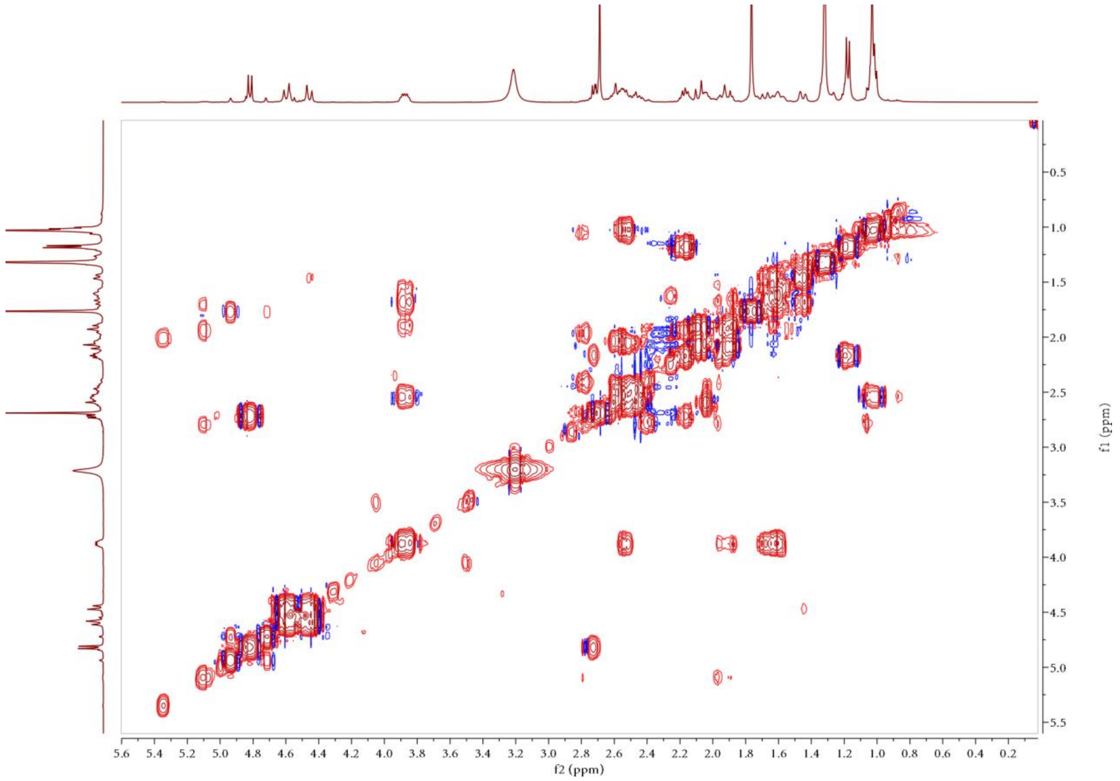

Figure S40. ^1H-^1H COSY ($CDCl_3$) spectrum of **7**

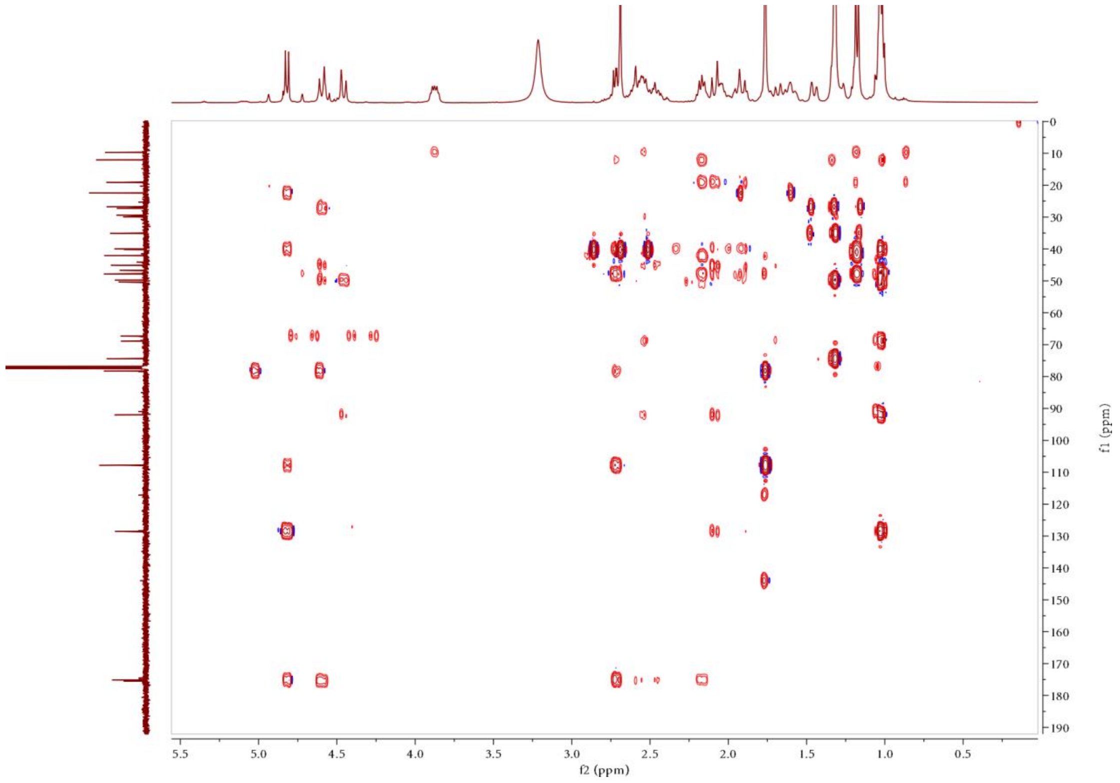

Figure S41. HMBC ($CDCl_3$) spectrum of **7**

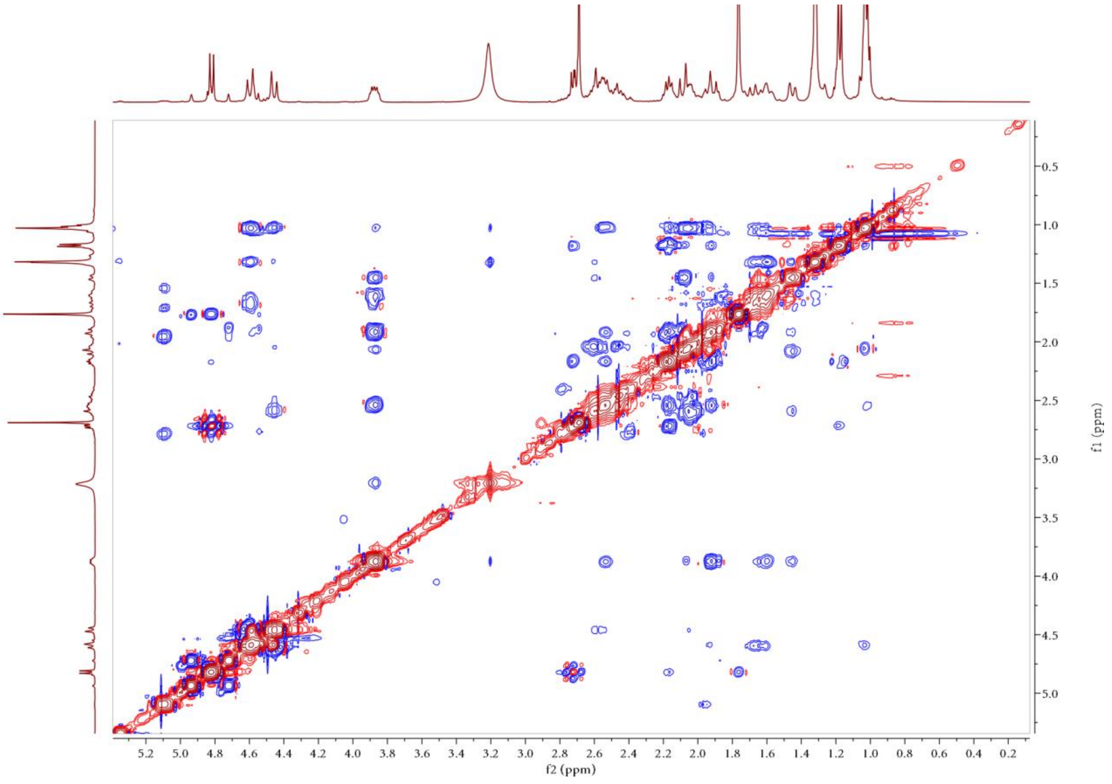

Figure S42. NOESY ($CDCl_3$) spectrum of **7**

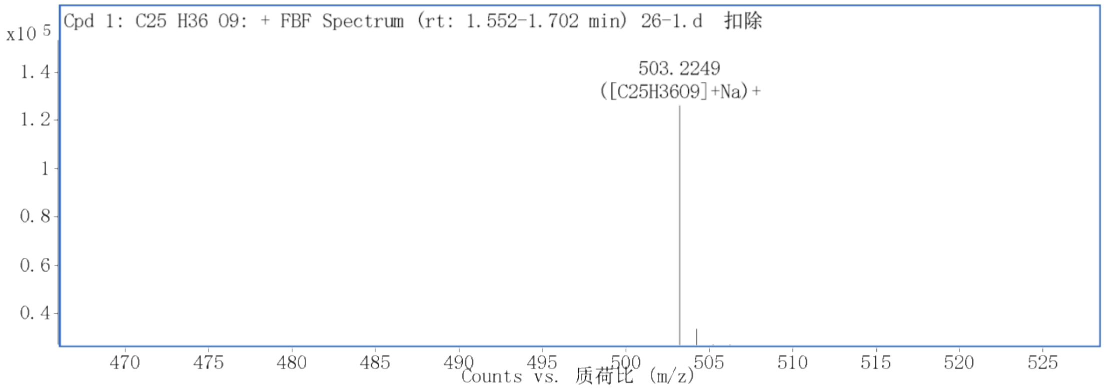

Figure S43. HRESIMS spectrum of **7**

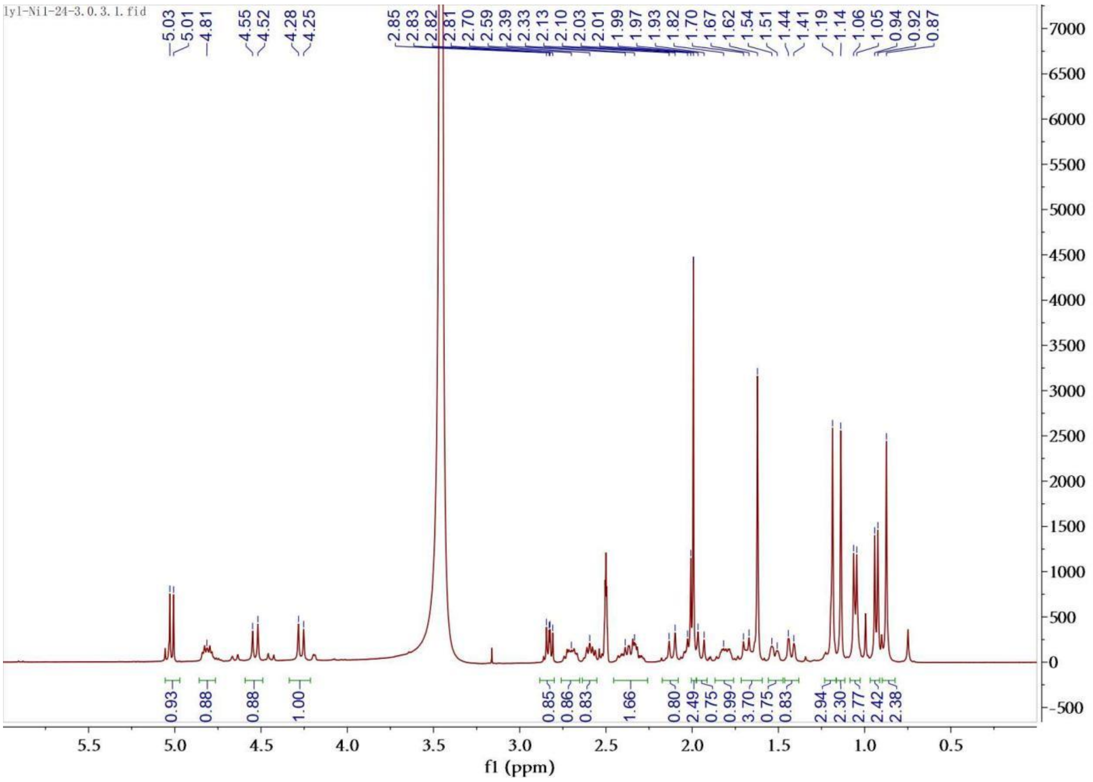

Figure S44. ^{1}H NMR (400 MHz, DMSO-d_6) spectrum of **8**

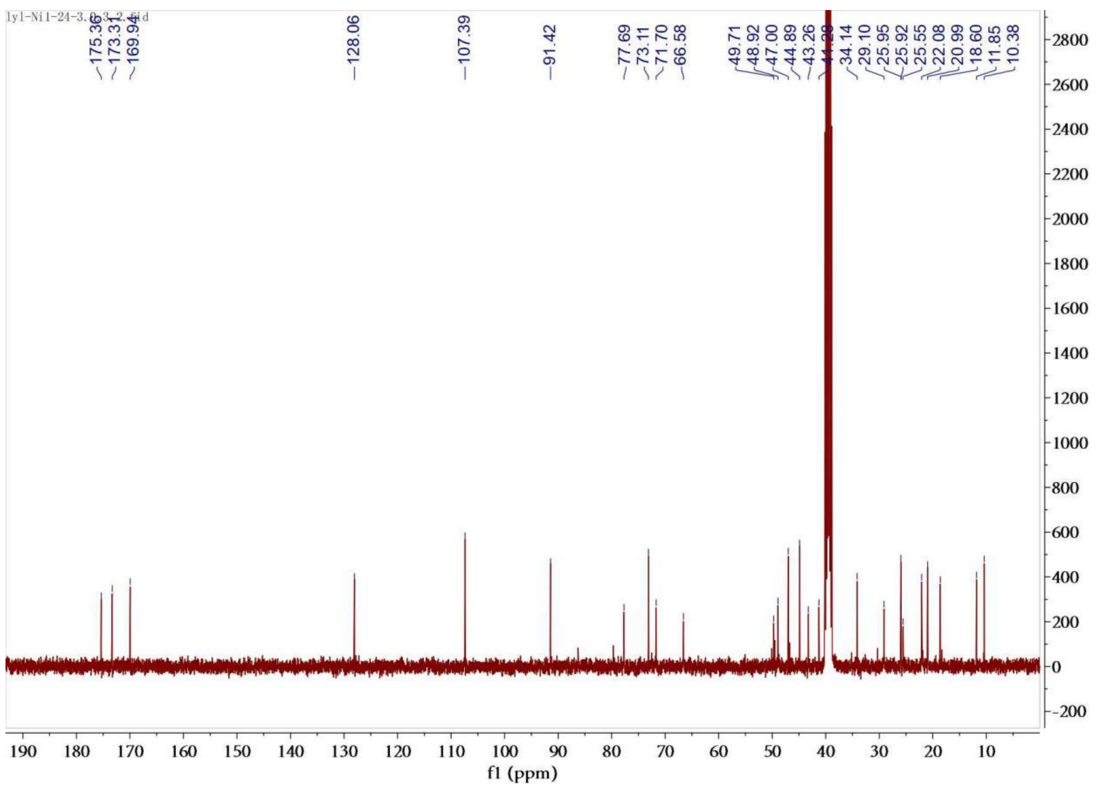

Figure S45. ^{13}C NMR (100 MHz, DMSO-d_6) spectrum of **8**

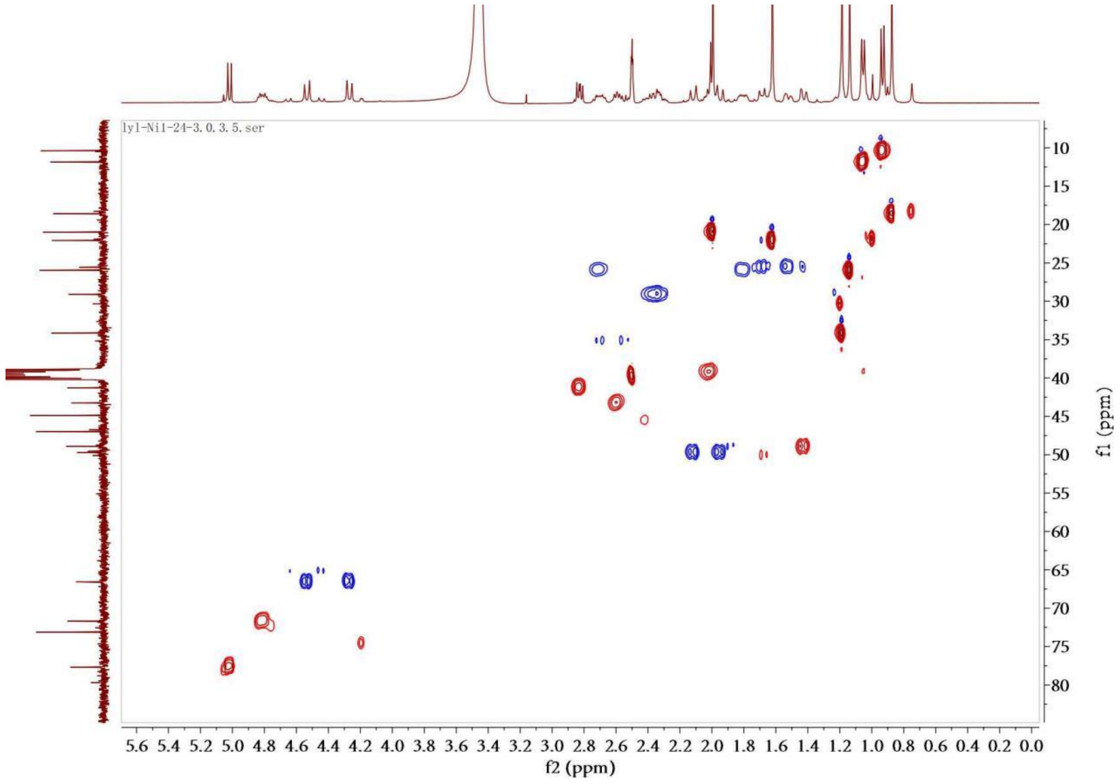

Figure S46. HMQC (DMSO-d_6) spectrum of **8**

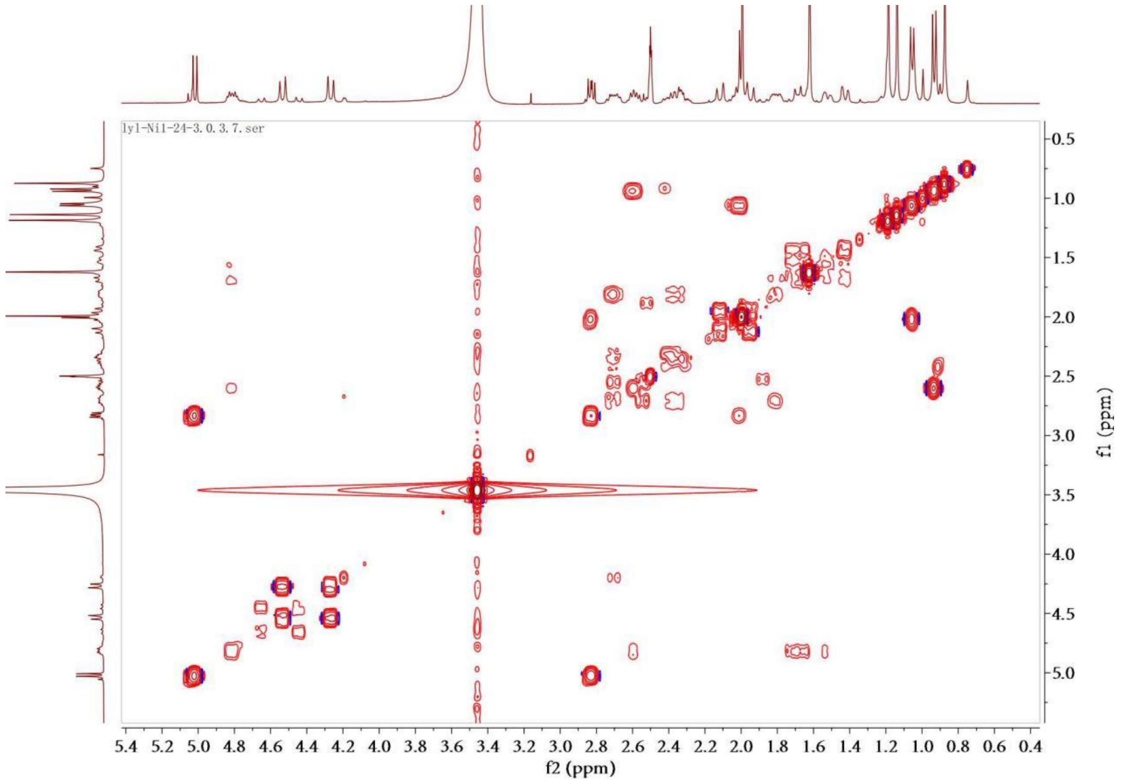

Figure S47. ^{1}H-^{1}H COSY (DMSO-d_6) spectrum of **8**

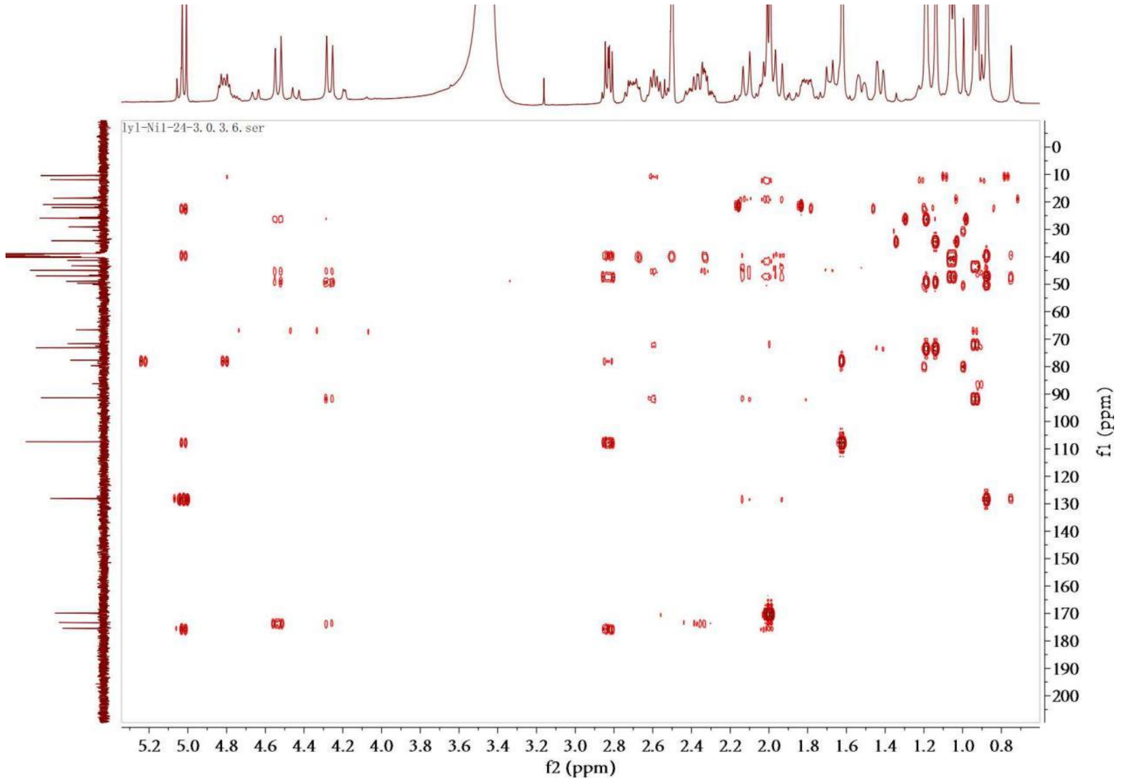

Figure S48. HMBC (DMSO-d_6) spectrum of **8**

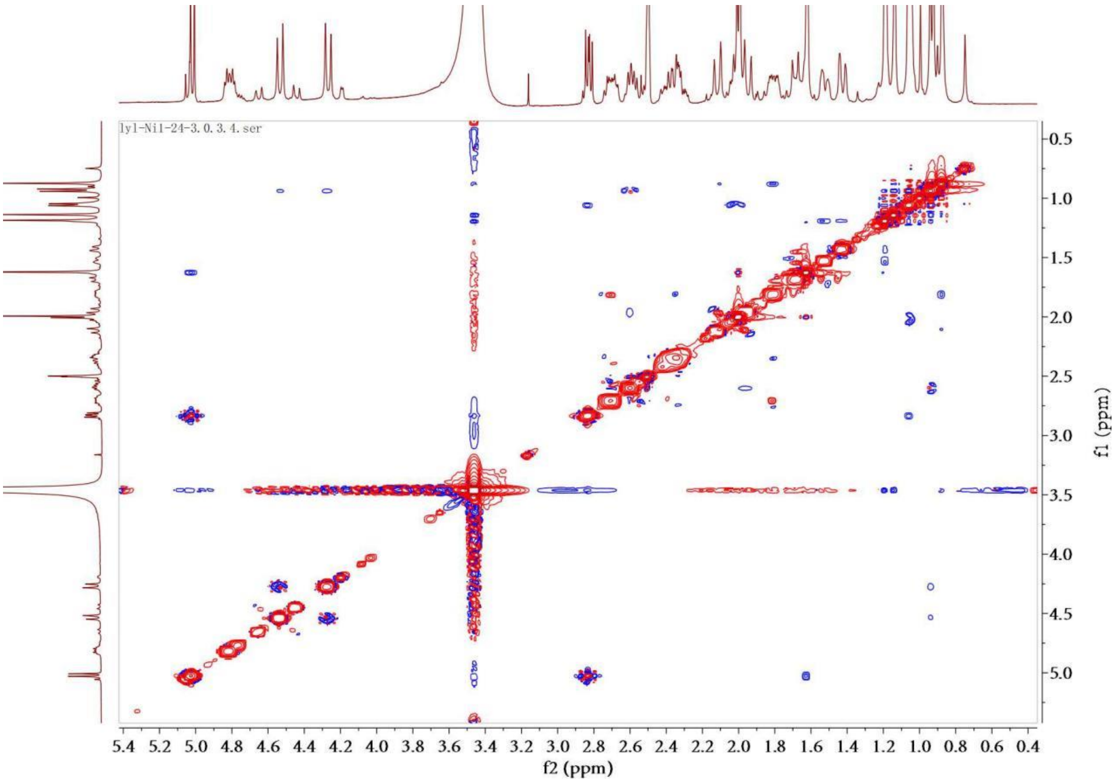

Figure S49. NOESY (DMSO-d_6) spectrum of **8**

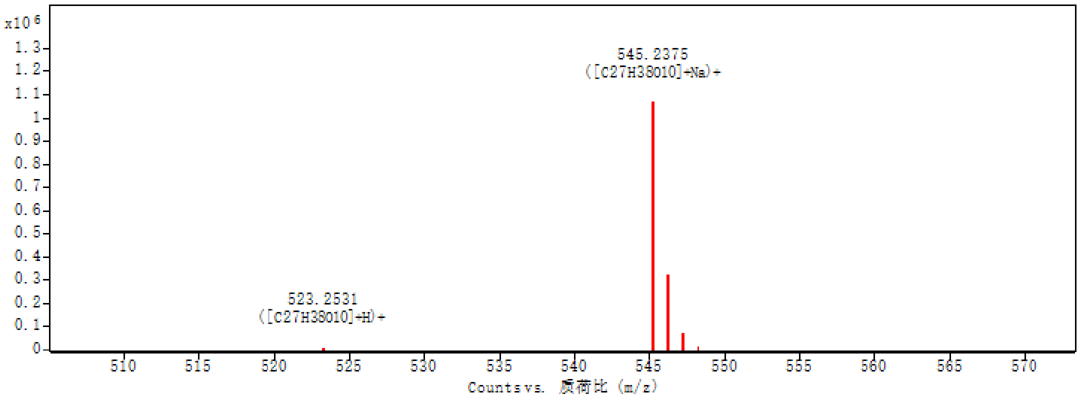

Figure S50. HRESIMS spectrum of **8**

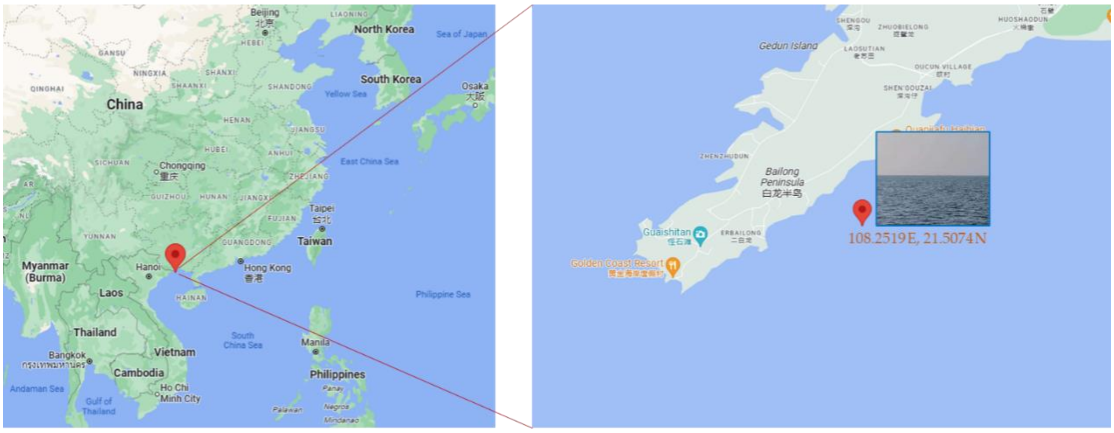

Figure S51. Map of location sites of sea sediment sample used in this study
